# Regularized CASPT2: an Intruder-State-Free Approach

**DOI:** 10.1021/acs.jctc.2c00368

**Published:** 2022-07-25

**Authors:** Stefano Battaglia, Lina Fransén, Ignacio Fdez. Galván, Roland Lindh

**Affiliations:** Department of Chemistry—BMC, Uppsala University, P. O. Box 576, SE-75123Uppsala, Sweden

## Abstract

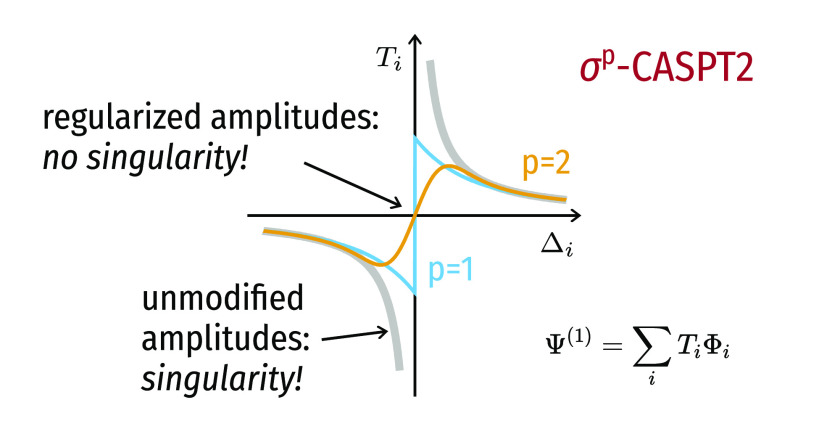

In this work we present a new approach to fix the intruder-state problem (ISP) in CASPT2 based on σ^*p*^ regularization. The resulting σ^*p*^-CASPT2 method is compared to previous techniques, namely, the real and imaginary level shifts, on a theoretical basis and by performing a series of systematic calculations. The analysis is focused on two aspects, the effectiveness of σ^*p*^-CASPT2 in removing the ISP and the sensitivity of the approach with respect to the input parameter. We found that σ^*p*^-CASPT2 compares favorably with respect to previous approaches and that different versions, σ^1^-CASPT2 and σ^2^-CASPT2, have different potential application domains. This analysis also reveals the unsuitability of the real level shift technique as a general way to avoid the intruder-state problem.

## 1 Introduction

Among the many options of multireference electron correlation methods,^1^ approaches based on second-order perturbation theory (PT2) with a multiconfigurational reference function offer an appealing compromise between accuracy and computational complexity. Their popularity is reflected by the large number of available flavors,^2−8^ which typically differ in the partitioning of the Hamiltonian, the many-electron basis used to express the first-order wave function, or the conditions to obtain the expansion coefficients. One of the most known and used of these multireference perturbation theory (MRPT) approaches is complete active space PT2 (CASPT2),^2^ whose development has seen some important activity in recent times, with a newly modified zeroth-order Hamiltonian (CASPT2-K),^9^ new quasi-degenerate variants (XDW-CASPT2 and RMS-CASPT2),^10,11^ reduced scaling implementations,^12−14^ and analytic nuclear energy gradients and derivative couplings.^15−22^ Despite its popularity and general applicability, CASPT2 suffers from an issue common to other MRPT-based approaches, the intruder-state problem (ISP). While this generally does not appear when modeling the ground state of small organic compounds, it is much more common in transition metal complexes and excited-states applications. Two techniques have been introduced during the years to avoid this issue in CASPT2, namely, the real and imaginary level shifts.^23,24^ In both cases, the idea is to add a uniform shift to the resolvent operator, avoiding the singularity that causes the ISP. As a consequence, these techniques introduce a dependence of the results on a user-defined parameter, which should ideally be minimal, especially in the absence of intruder states. In other words, the results for well-behaved cases should be as *insensitive* as possible to the value of the input parameter. This is because it is common that, for a molecular system, only a subset of electronic states or conformations is affected by the ISP but all of them are treated with the same intruder-state-removal technique. In principle, the results of well-behaved cases should not change as a function of the parameter, as they did not require any intervention in the first place. This is for instance typical in the calculation of vertical transition energies, where only one or a few states of the excitation manifold are plagued by ISPs, albeit imposing the use of the shift for all states. Furthermore, considering the current wide availability of analytic nuclear energy gradients for CASPT2,^15,16,20,21,25,26^ exploration of the potential energy surfaces, especially in the excited states,^27^ has become more common, requiring a robust approach that effectively avoids ISPs and that at the same time does not affect the qualitative description in regions of the PES where these are not present. In this context, the real level shift^23^ is not an ideal solution, the reason for which the imaginary level shift^24^ had been developed. This second option is much better, because it selectively corrects large amplitudes. Nevertheless, the results obtained remain susceptible to the choice of input parameter. In an attempt to find a new intruder-state-removal technique that is more insensitive to this, but equally effective in removing the singularities in the first-order wave function, we propose to use σ^*p*^ regularization in CASPT2.^28,29^ This approach is a simple and effective way to remove the ISP, and that from a theoretical perspective appears to have some advantages over the real and imaginary level shift techniques. In this contribution we introduce the σ^*p*^-CASPT2 method and critically analyze it in comparison to the established alternatives.

The work is organized as follows. In the next section we present the theoretical foundations of the ISP and the various intruder-state-removal techniques. We then conduct a series of tests to evaluate the effectiveness of σ^*p*^-CASPT2 in removing the ISPs and study the sensitivity of this new approach with respect to the input parameter. In the last section we summarize our findings and briefly discuss the remaining open issues in this context and possible future directions to solve them.

## 2 Theoretical Background

In this section we will define the intruder-state problem from a phenomenological perspective, review the theory behind the level shift techniques, and introduce the σ^*p*^ regularization formalism. The discussion is very general and applies, in principle, to any approach based on second-order Rayleigh–Schrödinger perturbation theory (RSPT2). However, in the following, these techniques are presented in the context of CASPT2, as this method is the focus of the present work.

### 2.1 Elements of Second-Order Perturbation Theory

The starting point is the partitioning of the Hamiltonian into a zeroth-order part and a perturbation operator (also known as fluctuation potential):
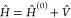
1The wave function of the reference state Ψ^(0)^ is an eigenfunction of *Ĥ*^(0)^ by construction (which does not necessarily have to be the ground-state one), with an associated energy eigenvalue *E*^(0)^. Note that in state-specific CASPT2, this energy does not correspond to the CASSCF one but rather to the expectation value of the generalized Fock operator (this is just as in MP2, where the zeroth-order energy does not correspond to the Hartree–Fock one). The first-order interacting space (FOIS) is spanned by an additional set of *M* eigenfunctions of *Ĥ*^(0)^, satisfying the following eigenvalue equation:
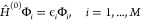
2In CASPT2, the functions Φ_*i*_ are (linear combinations of) internally contracted configurations generated by the application of excitation operators to the CASSCF reference wave function (eqs 1a–h in ref (2).), while in uncontracted theories such as MRMP2, they are configuration-state functions or Slater determinants.^3^ In either case, ϵ_*i*_ represents the zeroth-order energy associated with the *perturber* function Φ_*i*_. For the remainder of this article we will assume that the eigenfunctions Φ_*i*_ and eigenvalues ϵ_*i*_ are known, unless otherwise stated. This is in practice the case for the *diagonal* CASPT2 method^30^ (named CASPT2D in the original publication) but not for the (more conventional) full CASPT2 approach (referred to as CASPT2N by Andersson et al.^2^). In principle, it is possible to obtain the eigenpairs (Φ_*i*_, ϵ_*i*_) in CASPT2 as well, but this would require the diagonalization of *Ĥ*^(0)^ expressed in the FOIS basis, which is impractical from a computational perspective. Regardless of the particular implementation of RSPT2, the first-order correction to the wave function is expanded as follows,
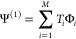
3where the amplitudes *T*_*i*_ are determined by solving the first-order equations:
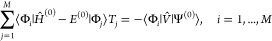
4Owing to eq 2 and the orthogonality of the eigenfunctions, the analytical expression for the amplitudes is simply
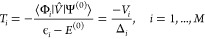
5where we have introduced a short-hand notation for the *right-hand side* elements *V*_*i*_ = ⟨Φ_*i*_|*V̂*|Ψ^(0)^⟩ and for the *energy denominators* Δ_*i*_ = ϵ_*i*_ – *E*^(0)^. At last, the second-order correction to the energy is obtained either by projection,
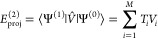
6or variationally, through the evaluation of the Hylleraas functional,^31^
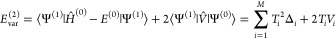
7Note that the solution of eq 4 is a stationary point of eq 7, hence the variational nature of the Hylleraas expression. Inserting that solution, i.e., eq 5, into either eq 6 or eq 7 results in the same second-order energy correction.

### 2.2 Intruder-State Problem

The *intruder-state problem* (ISP) arises when the energy denominator in eq 5 vanishes,
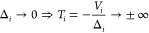
8leading to an infinitely large amplitude and a divergent perturbation series. This is caused by a degeneracy between Ψ^(0)^ and a perturber Φ_*i*_ in the zeroth-order approximation. The first obvious solution to this situation would be to include the intruder state Φ_*i*_ in the reference wave function. However, this is not always possible or desired in CASPT2, as it involves changing the active space of the underlying CASSCF optimization and potentially leads to the appearance of new intruder states, the need of additional electronic states in the state-averaging procedure, or exceedingly expensive calculations. The other option is to change the partitioning of the Hamiltonian. If the degeneracy between the intruder state Φ_*i*_ and Ψ^(0)^ is the result of the approximate description provided by the zeroth-order Hamiltonian, while the true energies of these states are different, one could modify the structure of *Ĥ*^(0)^ to lift this accidental degeneracy. In fact, the potential existence of an ISP is intimately coupled to the form of the zeroth-order Hamiltonian. For instance, an internally contracted approach based on Dyall’s Hamiltonian^32^ is known to be practically free from intruder states, as in the case of NEVPT2.^6,33,34^ Thus, it seems a natural choice to focus the efforts for solving the ISP in CASPT2 on modifying *Ĥ*^(0)^, analogously to the strategy followed by Roos and Andersson^23^ and Forsberg and Malmqvist^24^ several years ago, with the introduction of the real and imaginary level shift techniques, respectively. It is important to note that, even though from a formal point of view an intruder state strictly implies an exact degeneracy, in practice, all perturbers Φ_*i*_ associated with small energy denominators are considered intruder states. This is because the corresponding amplitudes will lead to artificially large contributions to the correlation energy. Rigorously speaking, it is possible that higher-order corrections compensate for that, though, for all practical purposes, this is not relevant, because the perturbation expansion is generally considered up to second-order only (for a formal discussion on the matter, we suggest the excellent work by Olsen and Jørgensen^35^ and references therein).

Before moving on to review the level shift techniques in CASPT2, it is important to introduce a convenient way to identify intruder states in actual calculations. Recognizing that an underlying assumption of perturbation theory is that the fluctuation potential *V̂* is a minor modification of the zeroth-order description of the system, it is expected that the first-order correction to the wave function is small compared to Ψ^(0)^. A way to quantify this is to consider the weight of Ψ^(0)^ relative to Ψ^(1)^ in the wave function corrected through first order, Ψ^[1]^ = Ψ^(0)^ + Ψ^(1)^. This gives rise to the diagnostic measure called *reference weight*, which is given by
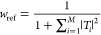
9Here, intermediate normalization is assumed (this is the case of CASPT2), thus assigning a unit coefficient to the reference wave function. In the trivial case where *w*_ref_ = 1, all amplitudes must be zero and thus the first-order correction does not contribute at all to Ψ^[1]^. On the other hand, increasingly small values of *w*_ref_ imply larger contributions to the total wave function from Ψ^(1)^; this is the typical situation. The limiting case *w*_ref_ = 0 is formally possible only in the presence of a *true* intruder state, where the amplitude of the offending state Φ_*i*_ diverges; *T*_*i*_ → ∞. In practice, accidental exact degeneracies almost never occur and small energy denominators are instead the typical cause of troubles, leading to large amplitudes that outweigh the reference wave function. This situation is associated with small values of *w*_ref_, which we shall recognize as a signature of the intruder-state problem. Clearly, the reference weight is an empirical diagnostic measure, and therefore there is no universal threshold that unambiguously classifies Ψ^[1]^ as affected by intruder states. Typical values of *w*_ref_ for small organic molecules range between 0.7 and 0.9, while transition metal complexes with many open-shell electrons can have lower values due to the dense manifold of electronic states and the presence of a large number of important low-lying configurations.

In any case, *w*_ref_ is expected to decrease with the number of correlated electrons regardless of the type of molecular system considered. Importantly, in most applications the interest is in relative energies, e.g., when computing vertical transition energies or comparing different conformations. For a balanced and consistent account of the electron correlation effects in these situations, it is crucial that the reference weight is commensurate among all structures and states considered. This simple guideline provides a prescription on how to use *w*_ref_ in practice. In calculations involving several electronic states, and in agreement with previous works,^9^ we consider as a rule of thumb deviations of more than 10% from the state with largest *w*_ref_, typically the ground state, as potentially damaged by intruder states. At the same time, the reference weight of any electronic state should be a smooth function of the molecular geometry, insomuch as the electronic energy is. This is particularly important in the calculation of potential energy surfaces and curves, such as for molecular dynamics simulations and the dissociation of diatomics.

### 2.3 Level Shift Techniques

With the realization that the intruder-state problem manifests itself when the energy denominator in eq 5 vanishes, a possible solution would be to add a small value ε > 0 to Δ_*i*_, such that when Δ_*i*_ → 0, *T*_*i*_ → −*V*_*i*_/ε. This is the simple idea behind level shift techniques. It can be formally implemented in any perturbation theory approach by modifying the partitioning of the Hamiltonian as follows:
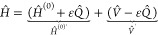
10where  is a projector that shifts only states orthogonal to the reference one. Solution of the first-order equations with this modified partitioning leads to amplitudes of the form
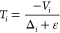
11For a vanishing energy difference, Δ_*i*_ → 0, the amplitude *T*_*i*_ now remains finite owing to the presence of ε in the denominator. This technique is very effective in removing intruder states from ground-state calculations, and it was proposed as a remedy to the ISP in CASPT2 by Roos and Andersson.^23^ However, because ε uniformly shifts all of the amplitudes of the first-order wave function, it affects also the coefficients of states Φ_*i*_ associated with large denominators, resulting in a second-order energy that strongly depends on it. In an attempt to decrease this dependence, a *level shift correction* that tries to minimize the impact of the shift on large denominators was proposed. The expression presented in the original contribution^23^ was derived from manipulating eq 6 and assuming that certain contributions are negligible in the case Δ_*i*_ ≫ ε; however, it was later found^24^ that this correction actually corresponds to simply evaluating the variational second-order energy, eq 7, using the modified amplitudes (see Section 1.1 in the Supporting Information for a detailed derivation). Unfortunately, the level shift approach with a real parameter does not really remove the singularity but rather moves it at Δ_*i*_ = −ε. While this is not an issue as long as ϵ_*i*_ ≥ *E*^(0)^, it is possible in CASPT2 that energy denominators take on negative values and accidentally fall near the singularity; that is, Δ_*i*_ ≈ −ε. This is particularly problematic when exploring several conformations, as it can be hard to find a suitable range for ε that avoids all singularities, or when computing excited states and investigating transition metals, where a large a number of small denominators make the results very sensitive to the value of the parameter.^36,37^

In an attempt at solving the shortcomings of the real level shift, Forsberg and Malmqvist^24^ proposed to use a purely imaginary parameter instead of a real one. In the *imaginary level shift* technique, the partitioning is simply modified as
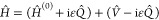
12which leads to complex amplitudes of the form
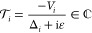
13To avoid working with complex arithmetic and considering that the electronic energy is a real number, only the real part of eq 13 is taken, resulting in the following expression for the imaginary-shifted amplitudes:
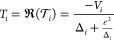
14Interestingly, this is essentially equivalent to the intruder-state avoidance technique developed for MRMP2^38^ and to Tikhonov regularization in linearized coupled cluster.^39^ In contrast to eq 11, eq 14 does not contain any singularity; hence, the imaginary level shift effectively removes the ISP. This happens by suppressing the contributions associated with small energy denominators: the smaller Δ_*i*_, the greater the reduction of *T*_*i*_. Vice versa, when Δ_*i*_ ≫ 0, we have that ε^2^/Δ_*i*_ → 0 and the amplitudes remain unmodified. Crucially, the ability of the imaginary shift to quench the contributions only when the energy denominators are small comes from its dependence on Δ_*i*_. As long as the zeroth-order Hamiltonian is diagonal, such as in CASPT2D and MRMP2, the values of these energy denominators are known. However, this is not the case for a nondiagonal *Ĥ*^(0)^, preventing the use of the *true* denominators in ε^2^/Δ_*i*_. In CASPT2, the imaginary shift is approximated by using only the diagonal entries of the zeroth-order Hamiltonian matrix, which typically yields a reasonable estimate of the exact Δ_*i*_ as long as the off-diagonal couplings are small. It is important to note that the imaginary shift is *in practice* a real shift too, as can be seen from eq 14. Its formal advantages over the real level shift technique of Roos and Andersson^23^ are not due to the use of complex algebra but rather to its *configuration-specific* nature, as opposed to the single uniform parameter appearing in the denominator of eq 11.

**Figure 1:**
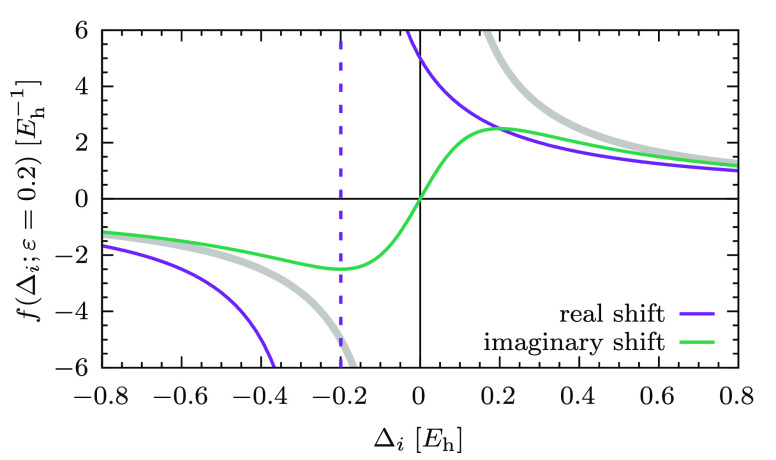
*f*(Δ_*i*_; ε = 0.2) for the real and imaginary level shifts as a function of the energy difference Δ_*i*_ = ϵ_*i*_ – *E*^(0)^. The gray thick line is the unmodified denominator with the singularity at the origin. The purple line is *f*(Δ_*i*_; ε = 0.2) for the real level shift (the dashed vertical line shows the position of the new singularity). The green line is *f*(Δ_*i*_; ε = 0.2) for the imaginary level shift.

At this point, it is convenient for the visual comparison of the two shifts, and for the discussion on the regularizers in the next subsection, to introduce the following general formula for the amplitudes:
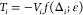
15where the function *f*(Δ_*i*_; ε) embodies the energy denominator and the different approaches that modify it (note the semicolon in eq 15 to highlight that ε is a fixed parameter). For instance, in the case of the real level shift, we have
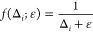
16while for the imaginary shift this is
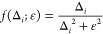
17Note how, in both cases, 1/Δ_*i*_ is recovered for ε = 0. The way in which the amplitudes are affected by the level shift technique is neatly captured by *f*(Δ_*i*_; ε), which is plotted in Figure 1 as a function of the energy denominator Δ_*i*_. As can be seen, *f*(Δ_*i*_; ε = 0.2) for the imaginary shift does not contain any pole, while the real one diverges at Δ_*i*_ = −0.2 *E*_h_.

Finally, note that also, in the case of the imaginary shift, it is possible to reduce the sensitivity of the energy with respect to ε by computing it using the variational expression given in eq 7.

### 2.4 σ^*p*^ Regularization

**Figure 2:**
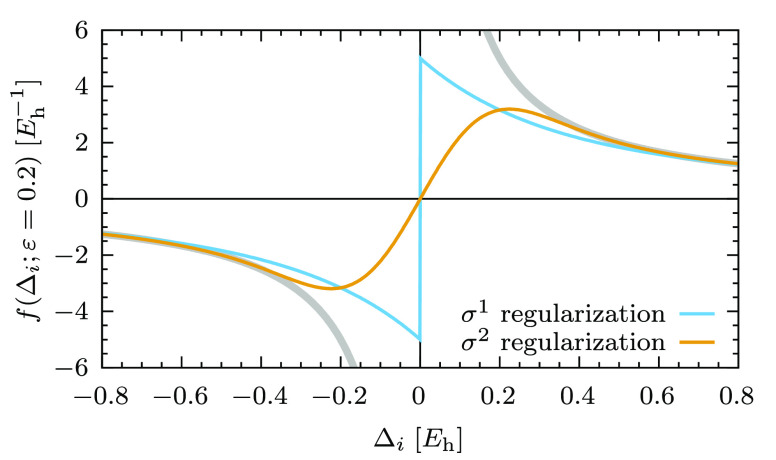
*f*(Δ_*i*_; ε = 0.2) for the σ^1^ and σ^2^ regularizers as a function of the energy difference Δ_*i*_. The light blue line is *f*(Δ_*i*_; ε = 0.2) for σ^1^, while the orange line, for σ^2^.

**Figure 3:**
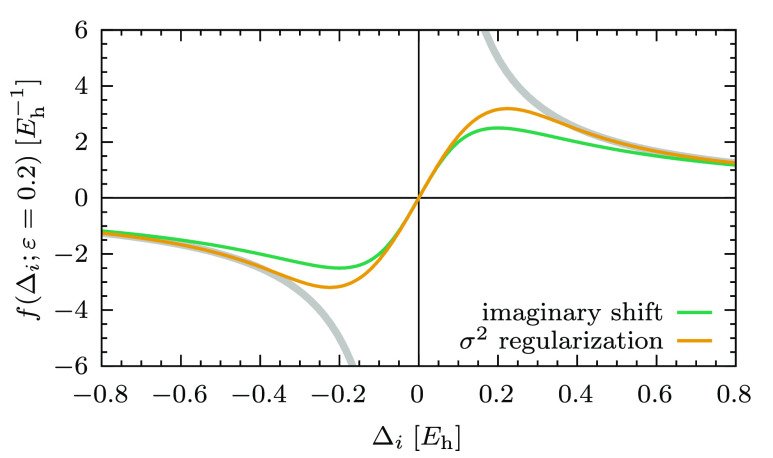
Comparison of *f*(Δ_*i*_; ε) for the imaginary level shift and σ^2^ regularization as a function of the energy difference Δ_*i*_. A value ε = 0.2 *E*_h_ was used for both cases.

An alternative approach to remove the singularity from eq 5 is the σ^*p*^ regularization technique introduced by Head-Gordon and co-workers in the context of (orbital-optimized) second-order Møller–Plesset perturbation theory (MP2).^28,29^ The theoretical foundation of σ^*p*^ regularization is rooted in the Laplace transform of the energy denominator, which is given by
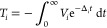
18When Δ_*i*_ = 0, the integrand is a constant function and *T*_*i*_ → ∞. A possibility to avoid such divergence is to change the upper limit of the integral to a value that is directly proportional to the energy difference Δ_*i*_, such that when Δ_*i*_ = 0 and the integrand is constant, the integral is bound by a finite upper limit. This strategy is implemented by the following expression, where, to accommodate for the possibility for negative denominators, we have introduced the absolute value and sign functions at appropriate places:
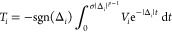
19In this equation, σ is a non-negative parameter and *p* is a positive number, which for simplicity we consider to be an integer value. The integration in eq 19 can be carried out analytically, resulting in the σ^*p*^-regularized amplitude:
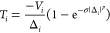
20For Δ_*i*_ → 0, (1 – e^–σ|Δ_*i*_|^*p*^^) goes to zero faster than 1/Δ_*i*_ diverges, such that the singularity is effectively suppressed. Similarly to the imaginary shift, the regularized amplitudes depend on the energy denominator appearing in the exponential factor, which in CASPT2 we approximate with the diagonal one as done for the imaginary shift. The integer value *p* changes the functional form of the regularizer and the way in which the singularity at Δ_*i*_ = 0 is avoided. In this work we consider the values *p* = 1 and *p* = 2, as these were found to be the most promising in regularized MP2.^29^ Interestingly, inserting eq 20 into eq 6 results in the same energy expression as in the driven similarity renormalization group approach.^40^ The parameter σ is the counterpart of ε in the real and imaginary shifts, and the two can be conveniently related by the following expression (see Section 1.2 in the Supporting Information for more details)
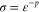
21In this way, we can define the regularizer in terms of ε in a manner similar to eqs 16 and 17, that is
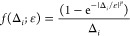
22The behavior of eq 22 for *p* = 1 and *p* = 2 as a function of Δ_*i*_ is shown in Figure 2. In both cases the singularity at the origin is removed. For *p* = 1 the amplitudes are damped to a finite value, thereby allowing each component of the first-order wave function to contribute to the correlation energy. On the other hand, the case with *p* = 2 is very similar to the imaginary level shift, where the amplitudes associated with small denominators are completely suppressed. In fact, it is instructive to directly compare *f*(Δ_*i*_; ε) for the imaginary shift and the σ^2^ regularizer, as shown in Figure 3. For matching values of the input parameter, the behavior at Δ_*i*_ → 0 is the same for both techniques. However, the σ^2^ regularizer follows more closely the unmodified 1/Δ_*i*_ function for larger values of Δ_*i*_. We thus expect the σ^2^-regularized energy to be less sensitive to the input parameter ε than the imaginary level shift, which is supported by a Taylor expansion of *f*(Δ_*i*_; ε) around Δ_*i*_ → 0 and ε → 0 (see section 1.3 in the Supporting Information for details). Moreover, the energy obtained with σ^1^-regularized amplitudes is expected to be even less affected by the value of ε, because no contributions to the energy are suppressed; rather they are just damped to a finite value. However, this comes at the price of a discontinuity at Δ_*i*_ = 0. This does not constitute a problem for calculations at a fixed conformation, but a change in the sign of a denominator during a smooth distortion of the molecular geometry will lead to a jump on the potential energy surface. For this reason, the σ^1^ and σ^2^ regularizers will likely have different potential applications.

### 2.5 A Note on the IPEA Shift

Another famous shift used in CASPT2 that requires some attention is the IPEA shift.^41^ While it is technically a *shift*, its nature is fundamentally different from the techniques discussed in this contribution. The IPEA shift acts on the generalized Fock operator expressed in the molecular orbital basis and was introduced to fix a systematic energy underestimation of open-shell electronic configurations with respect to closed-shell ones. While this has in general a positive effect on the ISP, that is the number of ISPs is generally smaller with the IPEA shift than without, it is not its intended use. The techniques discussed in this work act on the zeroth-order Hamiltonian expressed in the internally contracted basis spanning the FOIS. This *directly* targets the energies appearing in the denominators of eq 5, whereas the IPEA shift has a more complicated *indirect* effect on them due to the internal contraction formalism. In other words, the IPEA shift modifies the energies ϵ_*i*_ of eq 5 in nontrivial ways that depend on the generalized Fock eigenvalues. Given this difference, a detailed account of the IPEA shift is omitted from the current work.

## 3 Results and Discussion

In this section we present a series of calculations to evaluate the effectiveness of σ^*p*^ regularization in solving the intruder-state problem in CASPT2 and compare it to the level shift techniques. We have implemented the σ^1^ and σ^2^ regularizers in a development branch of OpenMolcas^42,43^ and note that they work with any quasi-degenerate variant of CASPT2 currently available in the package. In the following, we shall refer to CASPT2 with σ^*p*^ regularization simply as σ^*p*^-CASPT2.

### 3.1 Chromium Dimer

The dissociation of the chromium dimer has proven to be one of the most challenging systems for multireference quantum chemical methods, and it has been used to test a plethora of approaches.^9,25,44−55^ Due to one 4s and five 3d unpaired electrons in the ^7^S ground state of each atom, the chromium dimer has a formal sextuple bond, which requires at least an active space of 12 electrons in 12 orbitals for the description of its dissociation. One of the challenges in this molecular system is the large imbalance of the role that dynamic correlation plays at different internuclear distances. Around the equilibrium, there is a significant overlap between the compact 3d orbitals, and the presence of many electrons in such a small space requires an accurate description of short-range correlation effects. This can be achieved by including a large number of Slater determinants in the expansion of the wave function, leading to a sizable contribution of dynamic electron correlation in this region of the potential energy curve (PEC). On the contrary, a crude description of the Coulomb cusp results in a significant overestimation of the repulsion between the electrons, which explains why the CASSCF PEC is strongly repulsive at short internuclear distances (see Figure S1 in the Supporting Information). The situation is qualitatively and quantitatively different during dissociation, where only the more diffuse 4s orbitals overlap, thereby decreasing the importance and contribution of dynamical electron correlation. Another difficulty encountered in the description of the chromium dimer, which is common to transition metal systems, is the presence of a dense manifold of low-lying electronic states. This makes MRPT approaches, and in particular the CASPT2 method, very susceptible to intruder states. As a matter of fact, the Cr_2_ molecule has been the quintessential test of the intruder-state problem since the early days of CASPT2 and has been used as a representative system to highlight the effectiveness of both the real and imaginary level shift techniques.^23,24^ In this work we keep this tradition and assess the σ^*p*^ regularizers on the dissociation of Cr_2_ as well. In particular, we adopt the minimal 12 electrons in 12 orbitals active space in association with the cc-pwCV5Z basis set^56^ and include scalar relativistic effects through the second-order Douglas–Kroll–Hess Hamiltonian.^57,58^ The CASPT2 potential energy curve is computed for the lowest singlet state using the modified *g*_1_ zeroth-order Hamiltonian,^59^ which provides a qualitatively correct shape of the PEC. All energies are given relative to twice that of the single atom and no correction for the basis set superposition error was considered, as it does not affect the potential existence of intruder states. The variational energy expression, eq 7, has been used in all cases unless otherwise stated. As a reference, we report the experimental curve of Dattani^60^ in all of the plots, which predicts a dissociation energy of 1.66 eV. Note that the main focus of these calculations is not to obtain the best possible ab initio results reproducing the experimental data but rather to investigate the effectiveness of the regularizers in suppressing the intruder states.

**Figure 4:**
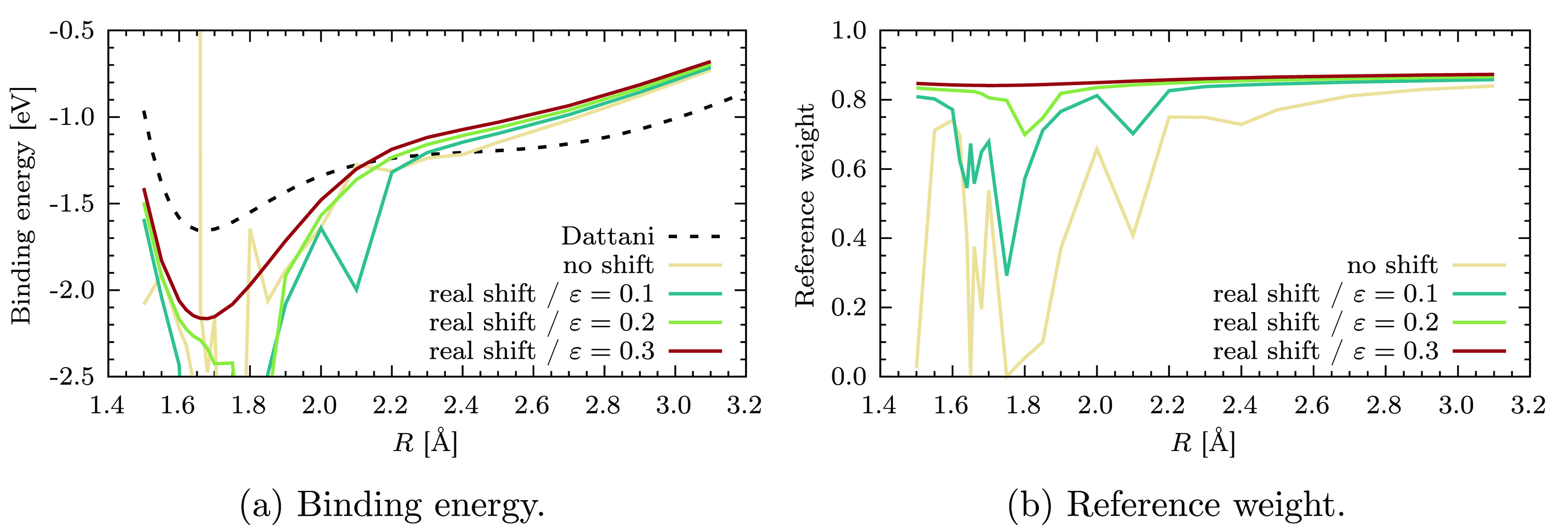
CASPT2 potential energy curves of Cr_2_ with and without using the real level shift (a) and corresponding reference weight *w*_ref_ (b) as a function of the internuclear distance *R*.

**Figure 5:**
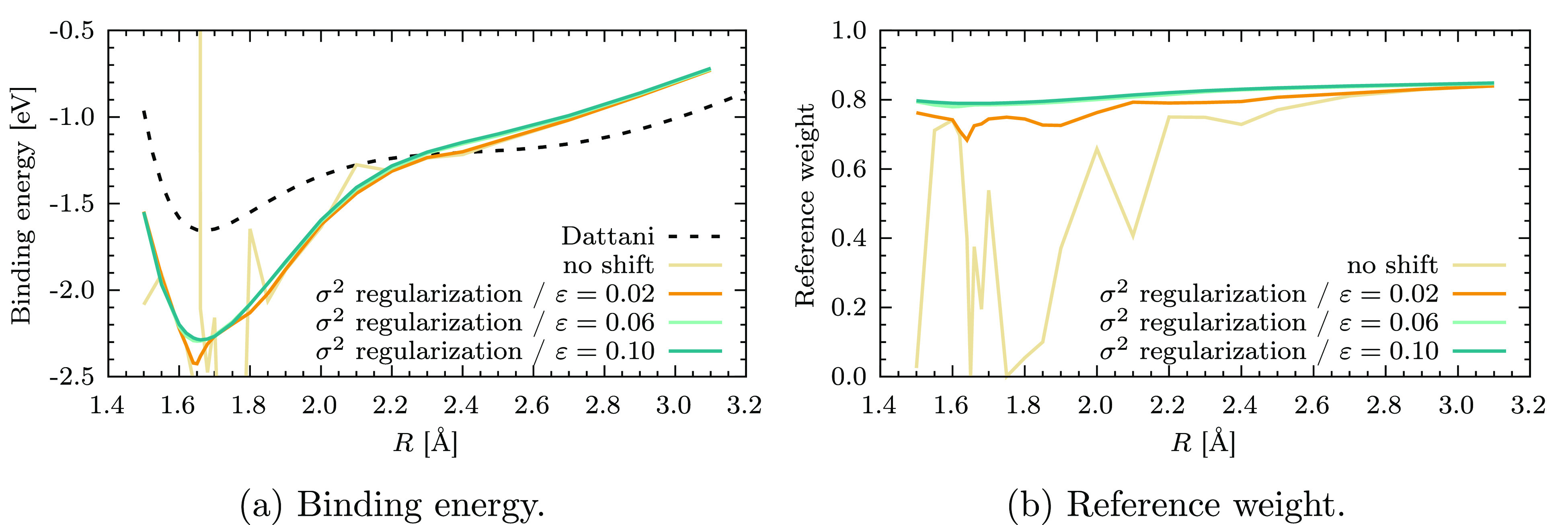
σ^2^-CASPT2 potential energy curves of Cr_2_ (a) and corresponding reference weights *w*_ref_ (b) as a function of the internuclear distance *R*.

**Figure 6:**
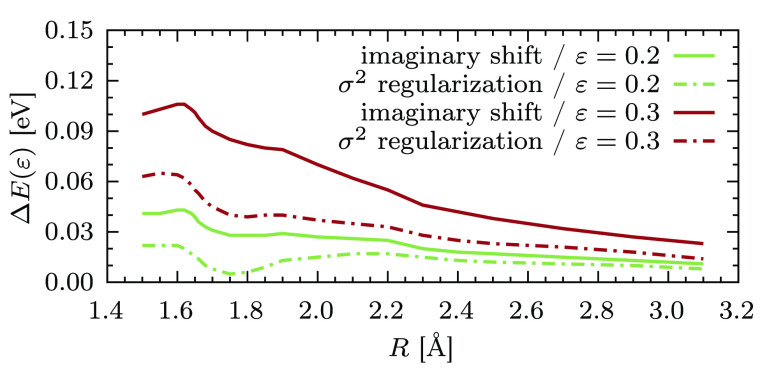
Energy differences Δ*E*(ε) = *E*(ε) – *E*_ref_ as a function of the internuclear distance *R*, for two different values of ε. The reference energy *E*_ref_ used for each technique was obtained with the value ε = 0.1 *E*_h_.

To this end, we start by showing in Figure 4a the CASPT2 PEC obtained with and without the real level shift, for an increasing value of ε. In the unmodified CASPT2 results, there are several places along the curve where the energy is clearly unphysical, and these are all associated with low reference weights, a typical signature of intruder states. This can be clearly seen from Figure 4b, where we plot *w*_ref_ as a function of the internuclear distance *R*. Due to the presence of negative denominators around Δ_*i*_ ≈ −0.1 *E*_h_, a level shift parameter of ε = 0.1 *E*_h_ is not sufficient to avoid all singularities. A value of ε = 0.2 *E*_h_ significantly improves the situation, though only at ε = 0.3 *E*_h_ the obtained potential energy curve is smooth. For this last case, *w*_ref_ varies only very little as a function of *R*, meaning that the first-order wave function provides a consistent correction at any point of the dissociation. Repeating the same systematic calculations with σ^2^-CASPT2 results in a smooth PEC with a much smaller value of ε, as can be seen from Figure 5a. In particular, already at ε = 0.06 *E*_h_ the reference weight is approximately constant throughout the dissociation, apart from a minor drop around *R* = 1.6 Å; see Figure 5b. At ε = 0.1 *E*_h_ both the binding energy and *w*_ref_ are extremely smooth functions of the internuclear distance, highlighting the effectiveness of σ^2^-CASPT2 in removing the intruder states, however, without requiring a large value of the regularization parameter. The curves shown in Figure 5a,b are essentially equivalent to those obtained with the imaginary shift, which we report in the Supporting Information (Figure S2a,b). Additional plots with other values of ε for both approaches confirm the comparable effectiveness of σ^2^-CASPT2 and the imaginary level shift technique (see Figures S2c–f and S3 in the Supporting Information). We analyze the sensitivity of the results with respect to the input parameter by computing the energy difference Δ*E*(ε) = *E*(ε) – *E*_ref_ throughout the dissociation for increasing values of ε. Considering the results obtained with ε = 0.1 *E*_h_ converged (that is, all intruder states are fully removed), we can set *E*_ref_ = *E*(ε = 0.1) and compute Δ*E*(ε = 0.2) and Δ*E*(ε = 0.3) for both techniques. These energy difference curves are shown in Figure 6. As elucidated by the theoretical discussion in the previous section, the second-order energy with imaginary level shift is more sensitive to the value of ε than the σ^2^-CASPT2 one. For instance, around *R* = 1.6 Å, the deviation from *E*_ref_ is almost double as much for the former approach for both values of ε considered.

However, these deviations are less than 1% of the total correlation energy introduced by the second-order correction.

**Figure 7:**
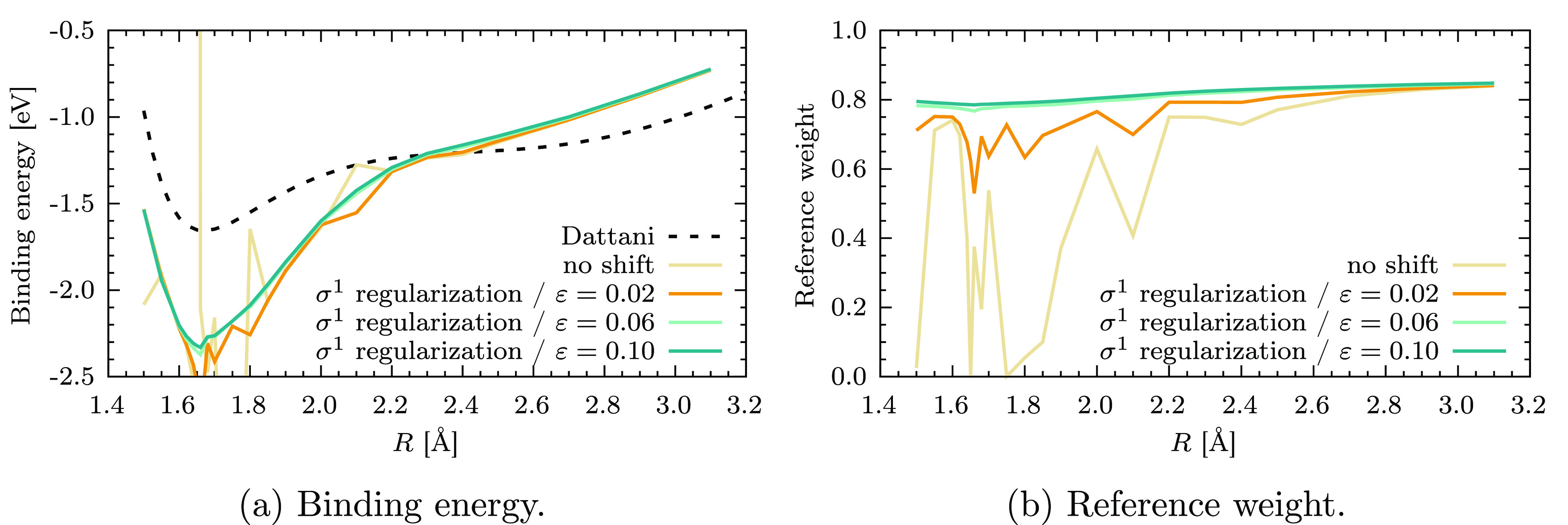
σ^1^-CASPT2 potential energy curves of Cr_2_ (a) and corresponding reference weights *w*_ref_ (b) as a function of the internuclear distance *R*.

The dissociation of the chromium dimer with σ^1^-CASPT2 is shown in Figure 7a, and the value of *w*_ref_ as a function of *R* is reported in Figure 7b. Also, in this case, ε = 0.1 *E*_h_ is sufficient to obtain a balanced first-order wave function throughout the dissociation and remove all intruder states. However, a noticeable jump in the energy is present near the equilibrium distance. This discontinuity is due to two perturbers, whose denominators flip sign from *R* = 1.66 Å to *R* = 1.68 Å. As a result, a net change of about 0.04 eV in the correlation energy happens between these two points, despite avoiding the singularity at Δ_*i*_ = 0. This is the issue alluded to earlier in the theoretical discussion of the σ^1^ regularizer, which limits its applicability to situations in which the molecular structure does not change. Note that, despite this potential problem, σ^1^-CASPT2 effectively removes the ISP and is very insensitive to the value of the regularization parameter ε (see Figures S4 and S5 in the Supporting Information).

Besides showing PECs with the smallest possible value of ε for which the ISPs are removed, we also report in Figure S6 of the Supporting Information the PECs obtained with artificially large values of ε in combination with all intruder-state-removal techniques. This highlights another aspect of these techniques, which is briefly discussed in the Conclusions section.

At last, we shall note that σ^*p*^-CASPT2 is less dependent on the expression used to obtain the energy, in contrast to the real and imaginary level shifts. For these, the difference between the energy obtained with eq 6 and eq 7—the level shift correction—is about 0.45 eV and 0.2 eV, respectively, for a level shift parameter ε = 0.3 *E*_h_. On the other hand, for σ^2^-CASPT2 and σ^1^-CASPT2 this is about 0.08 eV and 0.15 eV, respectively, for the same value of the input parameter ε = 0.3 *E*_h_ (see Figures S7–S12 in the Supporting Information).

### 3.2 Vertical Excitation Energies

The dissociation of the chromium dimer has shown that both the imaginary shift and σ^2^-CASPT2 are equally effective in removing the intruder-state problem and provide a smooth potential energy curve everywhere. This is the case for the real shift too, albeit with a relatively large value of the shift parameter, which potentially has a negative impact on the overall accuracy. On the other hand, Cr_2_ was the perfect example to evidence the main shortcoming of σ^1^-CASPT2—the discontinuity at Δ_*i*_ = 0—which limits its use for this type of applications even though it effectively removed the intruder states. We shall now turn our attention to calculations at a fixed molecular geometry, and in particular to a typical application of the CASPT2 approach: vertical transition energies. In the following, the objective is 2-fold. First, we evaluate the effect of σ^*p*^ regularization and the level shift techniques in cases where no intruder-state problems are present. In particular, we are interested in measuring the sensitivity of the results with respect to the input parameter and quantify to what extent these deviate from the unmodified CASPT2 energies. Ideally, the *best* technique affects these results the least, as in these situations a modification is not really needed. We call this *NOISP analysis*. Second, we test the effectiveness of these techniques in fixing the ISP. This case is similar to the chromium dimer dissociation, where removal of singularities was necessary to obtain physical results, though the context is different here, because we consider fixed molecular geometries. To this end, we investigate vertical excitation energies which are affected by intruder-state problems. We call this *ISP analysis*. To carry out these two analyses, we employ Thiel’s benchmark set^61^ and systematically investigate a large number of singlet and triplet vertical excitation energies in 28 small organic compounds. The geometries are taken from the original work and correspond to MP2-optimized structures, in combination with the 6-31G*^62,63^ basis set. The selection of active spaces and the number of states included in the SA-CASSCF optimization follow closely that of the original reference.^61^ The starting point for both analyses is the calculation of 311 singlet and triplet excitation energies using the MS-CASPT2 method^64^ (and its diagonal counterpart, MS-CASPT2D), in association with the TZVP basis set^65^ and the atomic compact Cholesky decomposition of the two-electron integrals^66^ (using the default threshold value of 10^–4^*E*_h_). Here, the IPEA shift^41^ was set to zero and the unmodified generalized Fock operator was used (i.e., no *g*_1_ zeroth-order Hamiltonian^59^). First, we performed these calculations without any shift or regularization, which resulted in 117 states with a reference weight deviating by more than 10% from the ground-state one (109 for MS-CASPT2D). These were considered affected by intruder states and hence excluded from the NOISP analysis. The MS-CASPT2 transition energies of the remaining 194 (202 for MS-CASPT2D) states form the set of reference values for the NOISP analysis, as they do not require any shift or regularization. The full set of 311 states is instead used in the ISP analysis. Please refer to Section S3.1 of the Supporting Information for the detailed account of which states are included in which analysis. In the NOISP analysis, we compare the results obtained with an increasing value of ε to the reference values (ε = 0). This provides a quantitative measure of the sensitivity of the excitation energies with respect to ε. Instead, in the ISP analysis we track the distribution of the reference weights as a function of ε. In particular, for each molecule we compute the relative deviation of the excited states *w*_ref_ (*w*_ref_^es^) with respect to the ground-state one (*w*_ref_^gs^); that is,
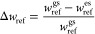
23(this is the same method used to identify the set of states affected by ISPs). An effective method fixing intruder-state problems should provide a distribution of Δ*w*_ref_ that quickly becomes narrow and peaked around zero for increasing values of ε. Instead of plotting the distributions directly, we compute the cumulative distribution function (CDF), which neatly captures how effective the different techniques are. For a given value of Δ*w*_ref_, the CDF essentially counts how many states have a reference weight deviation lower than Δ*w*_ref_ in proportion to the total number of states. Mathematically, this can be described as
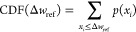
24where *p*(x_*i*_) is the (empirical) probability that there is a reference weight deviation *x*_*i*_ in the data set. The signature of an effective intruder-state-removal technique is a CDF function that quickly grows to 1.

From a theoretical perspective, we have seen in the previous section that the imaginary shift and σ^*p*^ regularization depend on the spectrum of the zeroth-order Hamiltonian. For a diagonal zeroth-order Hamiltonian, the use of any of these techniques strictly guarantees that intruder states are progressively removed by increasing the value of the input parameter. This is not the case for CASPT2, where the off-diagonal couplings in *Ĥ*^(0)^ could accidentally introduce intruder states. To provide an unbiased comparison between the shifts and the regularizers, we perform the NOISP and ISP analyses using the CASPT2D approach first. Nonetheless, we repeat the analyses using CASPT2 as well, as this is the method typically used in practice.

The root-mean-square and maximum absolute deviations (RMSD and MAD, respectively) of all techniques applied to CASPT2D for the NOISP set of energies are shown in Table 1. As it was the case for the chromium dimer, the results for the real level shift highlight its fundamental flaw; that is, the singularity is simply moved at a different place and not really removed. Even though among the 202 states considered here there are no small denominators in the unshifted calculation, increasing the value of ε has catastrophic effects. In a number of cases, negative denominators close to Δ_*i*_ ≈ −0.1 *E*_h_ and Δ_*i*_ ≈ −0.2 *E*_h_ cause divergences in the first-order wave function and lead to a very large root-mean-square deviation when ε = 0.1 *E*_h_ and ε = 0.2 *E*_h_. For instance, of the several hundred thousands of denominators appearing in the wave function expansion of the first ^1^B_1*g*_ and ^3^B_1*g*_ states of benzoquinone, two of them have a denominator equal to −0.103 *E*_h_ and −0.096 *E*_h_, respectively. These did not cause any issue for the reference calculations but led to ISPs for ε = 0.1 *E*_h_. The excitation energy obtained in these two cases is off by more than 8 eV, which is clearly incorrect. It is only with a larger value of ε = 0.3 *E*_h_ that no accidental ISPs are introduced by the real level shift. In the remaining lines of Table 1 we see a clear trend for the imaginary shift and the σ^*p*^ regularizers. First, no accidental intruder state appears in any of them, empirically proving the theoretical analysis carried out in the previous section. Importantly, for increasing values of ε, *w*_ref_ monotonically increases (see Figures S13–S15 in the Supporting Information for an example with the second ^1^B_2_ state of pyrimidine). Second, for matching values of ε, σ^*p*^-CASPT2 consistently shows smaller deviations from the reference energies compared to the imaginary level shift. Third, the RMSD and MAD increase at different rates as a function of ε for the three techniques. These results suggest that σ^*p*^-CASPT2 is less sensitive to the input parameter than CASPT2 with the imaginary shift. In particular, σ^1^ shows the least deviation, highlighting its potential in this scenario.

**Table 1:** NOISP Analysis for CASPT2D^a^

technique	ε = 0.1 *E*_h_	ε = 0.2*E*_h_	ε = 0.3*E*_h_
real	0.916 (9.80)	3.197 (45.3)	0.096 (0.39)
imaginary	0.013 (0.07)	0.029 (0.12)	0.052 (0.21)
σ^2^-reg	0.011 (0.07)	0.021 (0.10)	0.034 (0.12)
σ^1^-reg	0.008 (0.04)	0.017 (0.07)	0.029 (0.12)

a The values represent root-mean-square deviations (maximum absolute deviations in parentheses) of vertical transition energies obtained with different values of ε > 0 with respect to the reference ones obtained with ε = 0. All values are given in eV.

**Figure 8:**
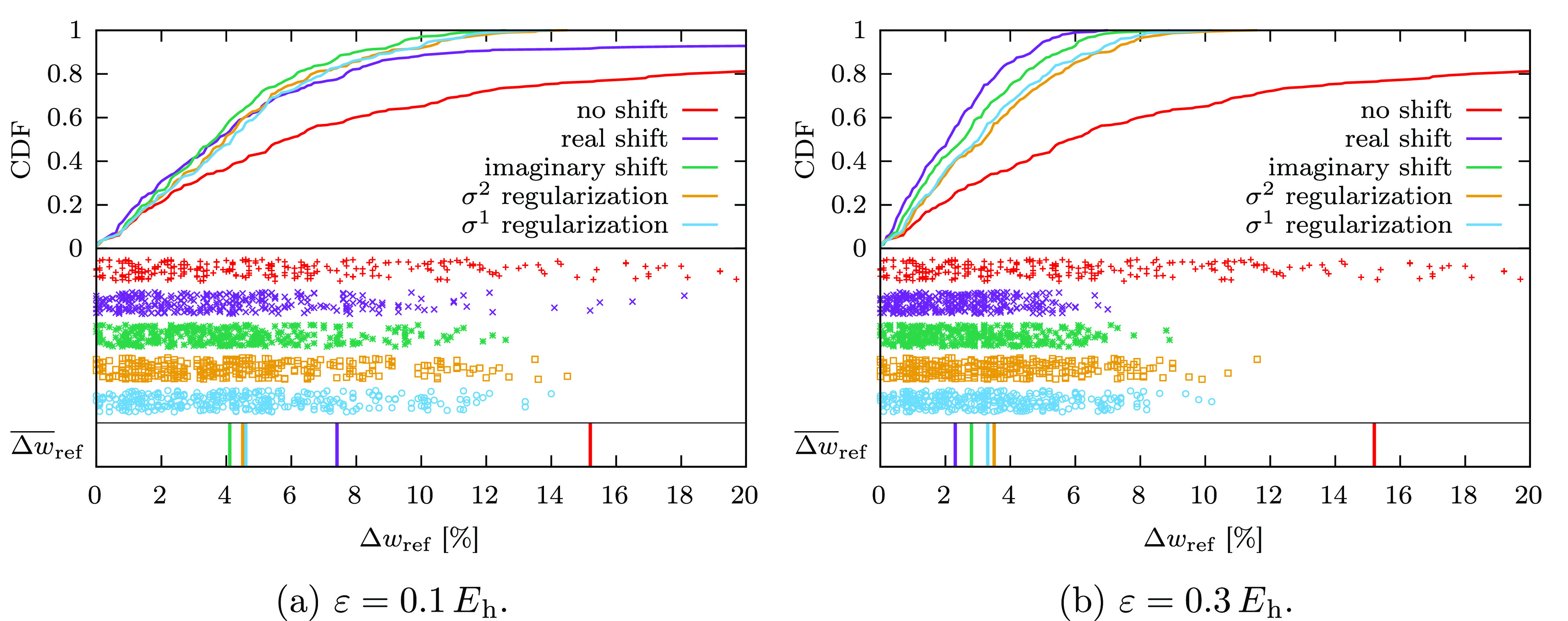
ISP analysis for CASPT2D. The cumulative distribution function is shown in the top half of the plots, while the values Δ*w*_ref_ < 20% are shown as points in the center of the plots. In the bottom stripe we show the position of the average reference weight deviation Δ*w*_ref_.

The situation is slightly different for the ISP analysis. Here we perform the calculation on the entire set that includes 311 excited states and track their reference weight deviations Δ*w*_ref_ with respect to the ground-state ones. Recall that, for ε = 0, there are 109 states that have a reference weight deviating by more than 10% from the ground-state one. In Figure 8a we plot the cumulative distribution function of Δ*w*_ref_ for ε = 0.1 *E*_h_ and ε = 0.3 *E*_h_, respectively (the plot for ε = 0.2 *E*_h_ is shown in the Supporting Information, Figure S19). For the unmodified calculation, one-fifth of the excited states obtained with the unmodified CASPT2D method have a Δ*w*_ref_ that is larger than 20% (note the CDF function at around 0.8 in the top right part of Figure 8a). Correspondingly, the first series of points shown in the center of Figure 8a—the red pluses—extends beyond the 20% mark (hence, it is not visible in the plot), resulting in a large average deviation. The real level shift improves this situation, producing a significantly more compact distribution of the Δ*w*_ref_ and a CDF that grows much faster in the range Δ*w*_ref_ < 10%. Nevertheless, several states remain affected by the ISP, which keep the average Δ*w*_ref_ relatively large. In contrast, the imaginary shift and σ^*p*^ regularization are much more robust, as evidenced by a CDF curve that reaches the value of 1 for Δ*w*_ref_ smaller than 15%. This means that no state of the 311 ones considered has a deviation of the reference weight larger than 15% with respect to the ground-state one. The distributions of the weights are very similar across the three approaches, with the imaginary shift slightly outperforming the regularizers, e.g., displaying a lower mean deviation of about 4% compared to ≈4.5% for σ^*p*^-CASPT2D. Increasing the value of ε to 0.3 *E*_h_ has a dramatic effect for the real level shift, which becomes the most effective way to fix the ISP, as evidenced by the fastest-growing CDF shown in Figure 8b. All intruder states are removed, and the average *w*_ref_ deviation is as low as ≈2%. The imaginary shift also improves significantly, with no deviation of the reference weights above the 10% mark. The smallest difference between ε = 0.1 *E*_h_ and ε = 0.3 *E*_h_ is obtained with σ^*p*^ regularization. This result is in agreement with the lower sensitivity with respect to the input parameter observed in the NOISP analysis.

Calculations using CASPT2D served to carry out an unbiased analysis of the various techniques; however, in practice the full CASPT2 approach is the method of choice. Hence, we repeated all of the calculations with the latter, investigating to what extent the off-diagonal couplings in the zeroth-order Hamiltonian affect the results obtained with CASPT2D. In Table 2 we show the RMSD and MAD obtained in the NOISP analysis with the CASPT2 method. The real shift is still plagued by accidental intruder states due to denominators that are close to the negative level shift parameter, and only for ε = 0.3 *E*_h_ there are no such cases. For the other three techniques, the results are almost identical to those in Table 1, and thus the same discussion holds. This is the case for the ISP analysis as well, with CASPT2 results virtually equal to CASPT2D ones; hence, we report the plots of the cumulative distribution functions in the Supporting Information (Figures S20–S22). As before, the real shift improves significantly upon increasing ε and shows the most compact distribution compared to the other techniques when ε = 0.3 *E*_h_. The imaginary shift and the σ^*p*^-CASPT2 differ only slightly and can be considered equally effective. It is interesting to note that, in a few cases, the off-diagonal couplings in the zeroth-order Hamiltonian have led to a *decreasingw*_ref_ for increasing values of ε, as illustrated for the second ^1^B_2_ state of pyrimidine in the Supporting Information (Figures S16–S18).

**Table 2:** NOISP Analysis for CASPT2^a^

technique	ε = 0.1 *E*_h_	ε = 0.2 *E*_h_	ε = 0.3 *E*_h_
real	0.866 (8.17)	1.765 (24.4)	0.095 (0.37)
imaginary	0.013 (0.06)	0.029 (0.12)	0.051 (0.21)
σ^2^-reg	0.010 (0.05)	0.020 (0.08)	0.033 (0.12)
σ^1^-reg	0.008 (0.03)	0.017 (0.06)	0.029 (0.12)

a The values represent root-mean-square deviations (maximum absolute deviations in parentheses) of vertical transition energies obtained with different values of ε > 0 with respect to the reference ones obtained with ε = 0. All values are given in eV.

Overall, the NOISP and ISP analyses suggest that the imaginary level shift and σ^*p*^ regularization have a comparable performance. On the one hand, the former is more sensitive to the level shift parameter but appears to be slightly more robust in solving the ISP. On the other hand, σ^*p*^-CASPT2 provides the smallest deviations from the reference energies in the NOISP analysis, in particular with *p* = 1. According to this latter result only, it appears that σ^1^-CASPT2 would be the best choice for calculations at a fixed molecular geometry. However, this comes with the inherent risk that small differences in the structures used (for instance obtained with a different methodology) might significantly affect the results in the unlucky case that one or more denominators change sign. Nevertheless, one can consider that already for ε = 0.1 *E*_h_ all ISPs are effectively removed by σ^*p*^-CASPT2 (and the imaginary shift), even though some states are above the (arbitrary) Δ*w*_ref_ = 10% threshold. We can see this by noting, e.g., that the energy differences are not unphysical and agree with values obtained with higher values of ε. After all, the reference weight is *one* possible measure to identify ISPs, and other criteria may be used as well, such as an analysis in terms of the excitation character of the perturbers with large contributions to the correlation energy. At last, the most striking result is for the real shift. A small value of ε is prone to generate accidental intruder states, but a large value, which is most effective in increasing the reference weight, is associated with the largest RMS deviations in the NOISP analysis. Remarkably, for all methods, no significant difference is observed between the CASPT2D and CASPT2 results.

## 4 Conclusions

In this work we have implemented the σ^*p*^-regularization technique in CASPT2. The resulting methodology, σ^*p*^-CASPT2, compares favorably to previous approaches based on the real and imaginary level shifts in terms of its efficacy in removing the intruder-state problem and the sensitivity of the results with respect to the input parameter. It was found that the two variants considered, σ^1^-CASPT2 and σ^2^-CASPT2, are suited for different use-case scenarios. σ^1^-CASPT2 is the least sensitive approach to the input parameter and effectively removes all intruder states for sufficiently large values of ε. However, due to the discontinuity of the regularized amplitudes at Δ_*i*_ = 0, its application is likely limited to calculations at a fixed molecular geometry, e.g., the determination of vertical transition energies. For calculations involving different molecular geometries, such as the dissociation of diatomic molecules or the exploration of potential energy surfaces, only the imaginary shift and σ^2^-CASPT2 ensure smooth results. This is because, in these two cases, the regularized amplitudes are a continuous function of the zeroth-order energy denominator. Both approaches remove all singularities equally well; however, σ^2^-CASPT2 is slightly less sensitive to the input parameter and, hence, our preferred choice. Nonetheless, we should note that the difference between them is probably insignificant compared to the overall accuracy offered by CASPT2 in the first place, such that in practice they both are valid options. Overall, the real level shift is the worst performer and should be avoided, due to its uniform action on all amplitudes. Only large values of the input parameter somewhat ensure no intruder states, but its results are the most sensitive to it, such that they may be considerably different from unshifted CASPT2 ones.

An important aspect related to σ^*p*^-CASPT2 and the imaginary level shift technique is their dependence on the energy denominator. From a strict theoretical perspective, these techniques are truly intruder-state-free only when used in combination with CASPT2D. In this case, increasing ε *always* leads to increased reference weights. This is not true for CASPT2, and there are cases in which the off-diagonal couplings of the Fock operator are such that accidental degeneracies appear for ε > 0. The use of the true eigenvalues of the CASPT2 *Ĥ*^(0)^ remains an open problem, and its solution is rather involved from the computational perspective because it would require the (at least partial) diagonalization of *Ĥ*^(0)^. On the positive side, the results obtained in this work for excitation energies suggest that this issue is statistically not so relevant.

It is also interesting to note that the level shift technique and σ^*p*^ regularization can be considered from a different point of view than that of intruder-state-removal approaches. In fact, for a given partitioning of the Hamiltonian, a renormalization of the first-order amplitudes can be interpreted as a way to introduce correlation effects from higher-order terms. This has the net impact of decreasing the amount of electron correlation captured by second-order perturbation theory, which is typically overestimated with respect to the infinite-order limit. Within this perspective, one could determine an optimal value for ε by comparing to experiment or high-level computational reference data, essentially defining a new separate methodology that is free of intruder states by design and potentially with a better accuracy. This is for instance the rationale behind regularized MP2 as proposed by Head-Gordon and co-workers,^29^ and what has been done with the intruder-state-avoidance technique in MRMP2.^67^ Nevertheless, the simple functional form of the shifts and regularizers cannot compensate the limitations inherent to a second-order perturbation theory framework. For instance, whereas a value of ε = 1.15 *E*_h_ for σ^2^-CASPT2 is such that the PEC of the chromium dimer matches the experimental one around the equilibrium, this comes at the cost of a far worse agreement at longer internuclear distances (see Figure S6 in the Supporting Information). This is because the effect of the regularizer is different at different correlation regimes, and there is no guarantee that a given value of ε is adequate everywhere. In light of this, our take on σ^*p*^-CASPT2 is for an approach that can be used routinely with a (small) fixed value of ε > 0 which fixes the intruder states, but that provides results as similar as possible to an unmodified version of CASPT2.

At last, we shall note that σ^*p*^ regularization can be used in combination with any variant of CASPT2 and RASPT2 currently implemented in the OpenMolcas package, and that its use does not pose a problem for the implementation of analytic nuclear energy gradients.

## Supporting Information Available

Additional formal derivations regarding level shifts and regularization; additional results on the chromium dimer; computational details and additional results on the ISP and NOISP analyses (PDF)
