# Supplementary material for: Regularized CASPT2: an Intruder-State-Free Approach
Source: J Chem Theory Comput. 2022 Jul 25;18(8):4814–25. doi: 10.1021/acs.jctc.2c00368 (PMC9367007; doi:10.1021/acs.jctc.2c00368)
Supplement: Supplementary file 1 — ct2c00368_si_001.pdf [file ct2c00368_si_001.pdf]

Supporting Information: Regularized CASPT2: an intruder-state-free approach

Stefano Battaglia,* Lina Fransén, Ignacio Fdez. Galván, and Roland Lindh*

Department of Chemistry - BMC, Uppsala University, P.O. Box 576, SE-75123 Uppsala, Sweden

E-mail: stefano.battaglia@kemi.uu.se; roland.lindh@kemi.uu.se

Contents

1 Theoretical background **S2**

1.1 Level-shift correction and variational energy expression S2

1.2 Relation of level shift and σ^p regularization parameters S4

1.3 Taylor expansion of level shifts and σ^p regularization S5

2 The chromium dimer **S7**

2.1 Potential energy curves S7

2.1.1 CASSCF S7

2.1.2 Imaginary shift S8

2.1.3 σ^2-CASPT2 . . . . S9

2.1.4 σ^1-CASPT2 . . . . S10

2.2 Sensitivity of σ^1-CASPT2 with respect to ε S10

2.3 Potential energy curves for large values of ε S11

2.4 Variational vs projected second-order energy S11

3 Vertical excitation energies **S14**

3.1 Computational details S14

3.2 ISP due to off-diagonal zeroth-order couplings S16

3.3 ISP analysis S19

References **S21**

1 Theoretical background

1.1 Level-shift correction and variational energy expression

The *level-shift correction* to the real level shift proposed in the original work by Roos and Andersson[1] was derived by considering the projected second-order energy expression (Equation (6) in the manuscript) with the level-shifted amplitudes (Equation (11) in the manuscript). Let us denote that quantity as $\tilde{E}^{(2)}_{\text{proj}}$, which reads

$$\tilde{E}^{(2)}_{\text{proj}} = \sum_{i=1}^{M} \tilde{T}_i V_i = -\sum_{i=1}^{M} \frac{V_i^2}{\Delta_i + \varepsilon} \tag{S1}$$

where $\tilde{T}_i$ are the shifted amplitudes (Equation (11) in the manuscript). We can manipulate Equation (S1) such that $\tilde{E}^{(2)}_{\text{proj}}$ is equal to the *unmodified* second-order energy plus a term

that depends on the level shift:

$$
\begin{aligned}
\tilde{E}^{(2)}_{\text{proj}} &= -\sum_{i=1}^{M} \frac{V_i^2}{\Delta_i + \varepsilon}\left(\frac{\Delta_i^2 + \Delta_i\varepsilon}{\Delta_i^2 + \Delta_i\varepsilon}\right) \\
&= -\sum_{i=1}^{M} \frac{V_i^2(\Delta_i^2 + 2\Delta_i\varepsilon - \Delta_i\varepsilon + \varepsilon^2 - \varepsilon^2)}{\Delta_i^3 + 2\Delta_i^2\varepsilon + \Delta_i\varepsilon^2} \\
&= -\sum_{i=1}^{M} \frac{V_i^2(\Delta_i + \varepsilon)^2 - V_i^2\Delta_i\varepsilon - V_i^2\varepsilon^2}{\Delta_i(\Delta_i + \varepsilon)^2} \\
&= -\sum_{i=1}^{M} \frac{V_i^2}{\Delta_i} - \sum_{i=1}^{M} \frac{-V_i^2\Delta_i\varepsilon - V_i^2\varepsilon^2}{\Delta_i(\Delta_i + \varepsilon)^2} \\
&= E^{(2)}_{\text{proj}} + \sum_{i=1}^{M} \frac{V_i^2\varepsilon}{(\Delta_i + \varepsilon)^2} + \frac{V_i^2\varepsilon^2}{\Delta_i(\Delta_i + \varepsilon)^2} \\
&= E^{(2)}_{\text{proj}} + \varepsilon\sum_{i=1}^{M} \frac{V_i^2}{(\Delta_i + \varepsilon)^2}\left(1 + \frac{\varepsilon}{\Delta_i}\right) \\
&= E^{(2)}_{\text{proj}} + \varepsilon\sum_{i=1}^{M} \tilde{T}_i^2\left(1 + \frac{\varepsilon}{\Delta_i}\right)
\end{aligned} \tag{S2}
$$

If there are no intruder states, we have $\Delta_i \gg \varepsilon$, such that $\varepsilon/\Delta_i \approx 0$ can be assumed in Equation (S2), resulting in:

$$
\tilde{E}^{(2)}_{\text{proj}} \approx E^{(2)}_{\text{proj}} + \varepsilon\sum_{i=1}^{M} \tilde{T}_i^2 \tag{S3}
$$

or, in rearranged form as

$$
E^{(2)}_{\text{proj}} \approx \tilde{E}^{(2)}_{\text{proj}} - \varepsilon\sum_{i=1}^{M} \tilde{T}_i^2 \equiv \tilde{E}^{(2)}_{\text{corr}} \tag{S4}
$$

Equation (S4) is the *level-shift corrected* second-order energy expression originally proposed by Roos and Andersson[1], which we denote here as $\tilde{E}^{(2)}_{\text{corr}}$. Note that Equation (S4) can also be seen in terms of the reference weight as defined on Equation (9) on the main manuscript, that is

$$
\tilde{E}^{(2)}_{\text{corr}} = \tilde{E}^{(2)}_{\text{proj}} - \varepsilon\left(\frac{1}{w_{\text{ref}}} - 1\right) \tag{S5}
$$

Importantly, it was later realised that Equation (S4) is equivalent to evaluating the variational second-order energy with the modified amplitudes $\tilde{T}_i$, but not with the modified $\hat{H}^{(0)}$

containing the shift. This is shown in the following:

$$
\begin{aligned}
\tilde{E}^{(2)}_{\text{var}} &= \sum_{i=1}^{M} \tilde{T}_i^2 \Delta_i + 2\tilde{T}_i V_i \\
&= \sum_{i=1}^{M} \frac{V_i^2 \Delta_i}{(\Delta_i + \varepsilon)^2} + \frac{-2V_i^2}{\Delta_i + \varepsilon} \\
&= \sum_{i=1}^{M} \frac{V_i^2 \Delta_i}{(\Delta_i + \varepsilon)^2} - \frac{V_i^2}{\Delta_i + \varepsilon} - \sum_{i=1}^{M} \frac{V_i^2}{\Delta_i + \varepsilon} \\
&= \tilde{E}^{(2)}_{\text{proj}} + \sum_{i=1}^{M} \frac{V_i^2 \Delta_i}{(\Delta_i + \varepsilon)^2} - \frac{V_i^2(\Delta_i + \varepsilon)}{(\Delta_i + \varepsilon)^2} \\
&= \tilde{E}^{(2)}_{\text{proj}} + \sum_{i=1}^{M} \frac{-V_i^2 \varepsilon}{(\Delta_i + \varepsilon)^2} \\
&= \tilde{E}^{(2)}_{\text{proj}} - \varepsilon \sum_{i=1}^{M} \frac{V_i^2}{(\Delta_i + \varepsilon)^2} \\
&= \tilde{E}^{(2)}_{\text{proj}} - \varepsilon \sum_{i=1}^{M} \tilde{T}_i^{\,2} \\
&= \tilde{E}^{(2)}_{\text{corr}}
\end{aligned}
\tag{S6}
$$

We can understand this result in the following way. For a given set of amplitudes, the variational expression gives the best possible estimate of the second-order energy correction when evaluated with the unmodified zeroth-order Hamiltonian. We can take advantage of this property and always use $\tilde{E}^{(2)}_{\text{corr}}$ to compute the energy associated to *any* type of modified amplitudes, that is, obtained with whichever shift or regularization technique.

1.2 Relation of level shift and σ^p regularization parameters

It is easiest to consider the limit the imaginary shift and the σ^2 regularizer when $\Delta_i \to 0$. This will ensure that both methods suppress the small energy denominators in the same way. The limit of the derivative of the imaginary shift is

$$
\lim_{\Delta_i \to 0} \frac{\partial}{\partial \Delta_i} \frac{\Delta_i}{(\Delta_i + \varepsilon)^2} = \frac{1}{\varepsilon^2} \tag{S7}
$$

while that for the σ^2 regularizer is

$$\lim_{\Delta_i \to 0} \frac{\partial}{\partial \Delta_i} \frac{1 - e^{-\sigma |\Delta_i|^2}}{\Delta_i} = \sigma \tag{S8}$$

which leads to the simple relationship $\sigma = \varepsilon^{-2}$.

In the case of the real level shift and σ^1 regularization we cannot use the same approach, as the derivative at $\Delta_i = 0$ is undefined. Instead, a simple dimensional analysis shows that ε for the real level shift must have energy units, as it enters directly the denominators. On the other hand, σ multiplies the energy denominator in the exponential factor of the regularizer, which should result in an overall dimensionless quantity, hence it must have units of inverse energy. We thus find the relationship $\sigma = \varepsilon^{-1}$ in the case of the σ^1 regularizer (which is taken with the arbitrary choice of a unit constant factor).

1.3 Taylor expansion of level shifts and σ^p regularization

It is instructive to expand $f(\Delta_i; \varepsilon)$ for the shifts and regularizers around $\varepsilon \to 0$ and $\Delta_i \to 0$. The former corresponds to the situation where $\Delta_i \gg \varepsilon$, hence the large denominator limit, while the latter to $\Delta_i \ll \varepsilon$, thus the small denominator limit. Let us first compare the imaginary level shift and the σ^2 regularizer, which share the same overall shape. For the former we have

$$\frac{\Delta_i}{\Delta_i^2 + \varepsilon^2} \approx \begin{cases} \dfrac{1}{\Delta_i} - \dfrac{\varepsilon^2}{\Delta_i^3} + \dots & , \varepsilon \to 0 \\ \dfrac{\Delta_i}{\varepsilon^2} - \dfrac{\Delta_i^3}{\varepsilon^4} + \dots & , \Delta_i \to 0 \end{cases} \tag{S9}$$

while for the latter we have

$$\frac{1 - \mathrm{e}^{-\Delta_i^2/\varepsilon^2}}{\Delta_i} \approx \begin{cases} \dfrac{1}{\Delta_i} - \dfrac{\mathrm{e}^{-\Delta_i^2/\varepsilon^2}}{\Delta_i} + \dots & , \varepsilon \to 0 \\ \dfrac{\Delta_i}{\varepsilon^2} - \dfrac{\Delta_i^3}{2\varepsilon^4} + \dots & , \Delta_i \to 0 \end{cases} \tag{S10}$$

In the large denominator limit, that is $\varepsilon \to 0$, we notice that the first term after $1/\Delta_i$ dies off more slowly for the imaginary shift than for the regularizer. This helps us understand why, for larger values of the denominator, the curve for the σ^2 regularizer more closely follows that of $1/\Delta_i$ than the one of the imaginary shift does (see Figure 3 in the manuscript). On the other hand, in the small denominator limit, that is $\Delta_i \to 0$, the two approaches are asymptotically the same, which is confirmed by their same behavior around the origin.

We can compare the real level shift and the σ^1 regularizer in a similar manner, which also have a similar overall shape (assuming $\Delta_i \geq 0$). For the real level shift we have

$$\frac{1}{\Delta_i + \varepsilon} \approx \begin{cases} \dfrac{1}{\Delta_i} - \dfrac{\varepsilon}{\Delta_i^2} + \dots & , \varepsilon \to 0 \\ \dfrac{1}{\varepsilon} - \dfrac{\Delta_i}{\varepsilon^2} + \dots & , \Delta_i \to 0 \end{cases} \tag{S11}$$

While for the σ^1 regularizer we have

$$\frac{1 - \mathrm{e}^{-|\Delta_i/\varepsilon|}}{\Delta_i} \approx \begin{cases} \dfrac{1}{\Delta_i} - \dfrac{\mathrm{e}^{-|\Delta_i/\varepsilon|}}{\Delta_i} + \dots & , \varepsilon \to 0 \\ \dfrac{1}{\varepsilon} - \dfrac{\Delta_i}{2\varepsilon^2} + \dots & , \Delta_i \to 0 \end{cases} \tag{S12}$$

The same discussion as above holds when comparing these two approaches. The limit $\varepsilon \to 0$ highlights while the σ^1 regularizer follows more closely the $1/\Delta_i$ function, while the $\Delta_i \to 0$ limit is equivalent for both methods.

2 The chromium dimer

2.1 Potential energy curves

2.1.1 CASSCF

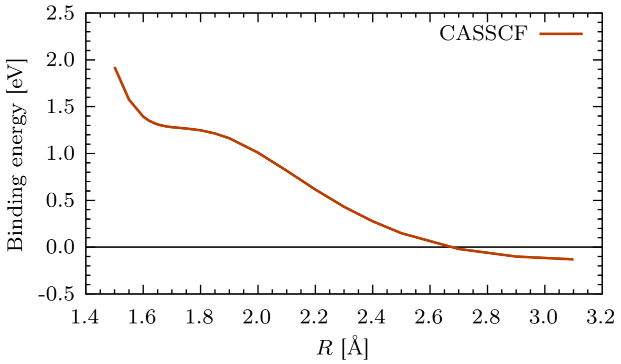

Figure S1: CASSCF potential energy curve of Cr_2 as a function of the inter-nuclear distance R.

2.1.2 Imaginary shift

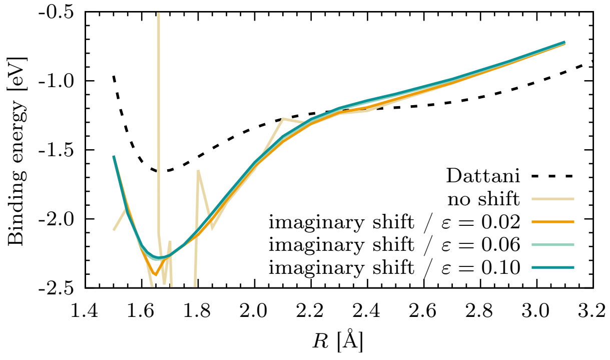

(a) Binding energy.

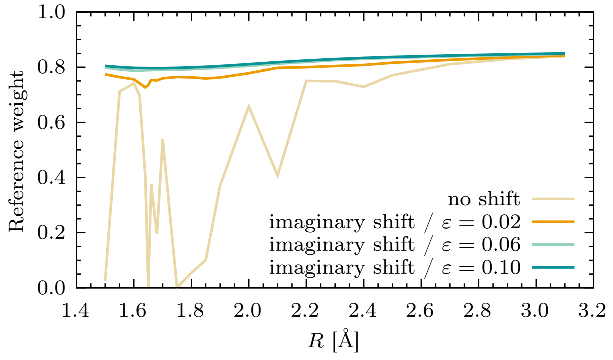

(b) Reference weight.

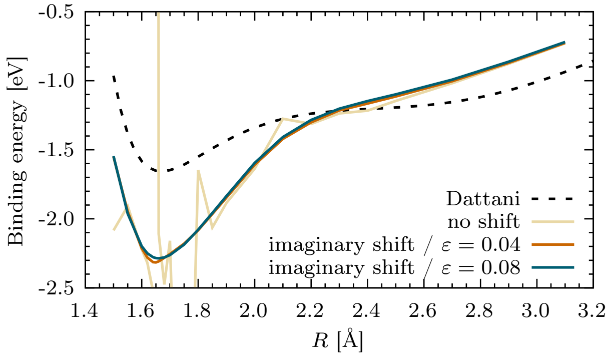

(c) Binding energy.

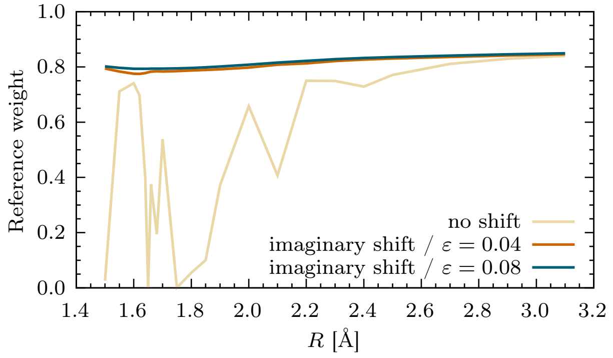

(d) Reference weight.

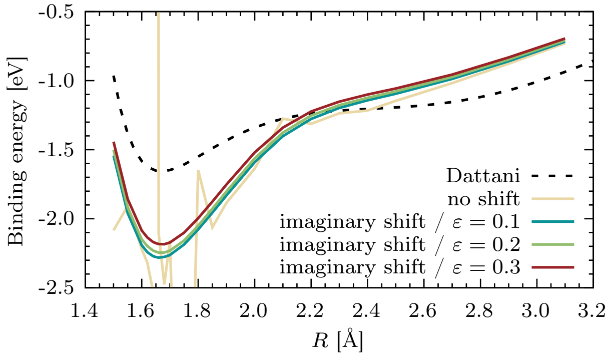

(e) Binding energy.

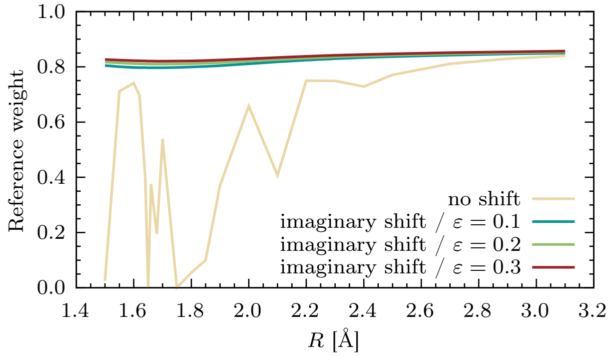

(f) Reference weight.

Figure S2: CASPT2 potential energy curve of Cr_2 with imaginary level shift for different range of ε (a, c and e), and corresponding reference weight w_{ref} (b, d and f) as a function of the inter-nuclear distance R.

2.1.3 σ^2-CASPT2

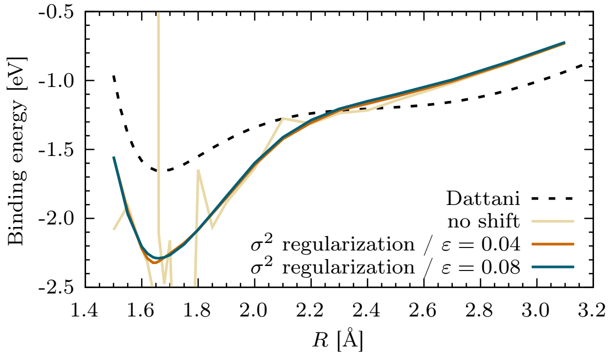

(a) Binding energy.

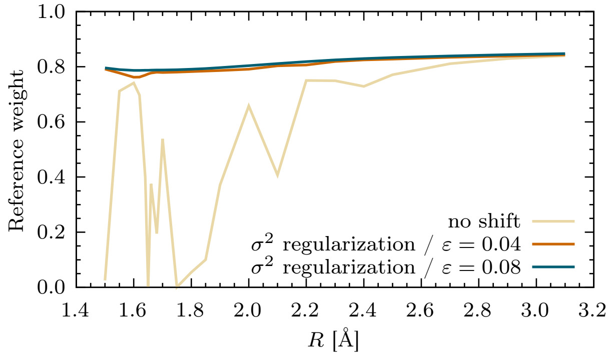

(b) Reference weight.

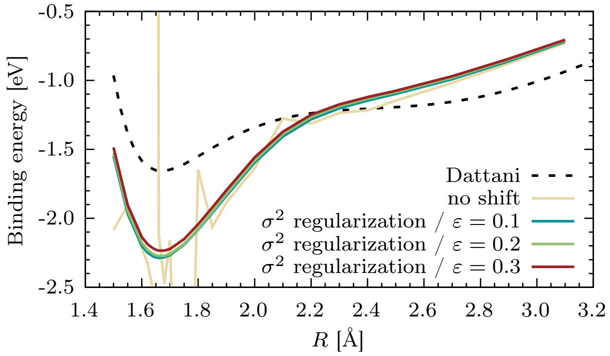

(c) Binding energy.

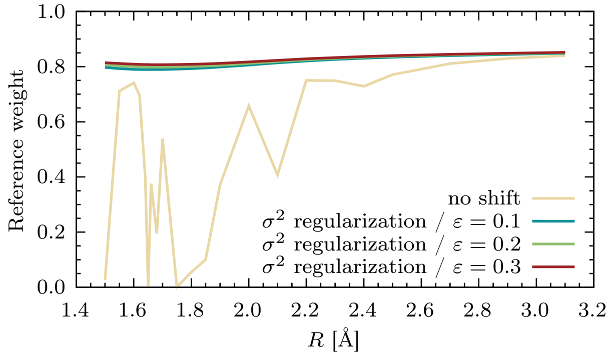

(d) Reference weight.

Figure S3: σ^2-CASPT2 potential energy curve of Cr_2 for different range of ε (a and c), and corresponding reference weight w_{ref} (b and d) as a function of the inter-nuclear distance R.

2.1.4 σ^1-CASPT2

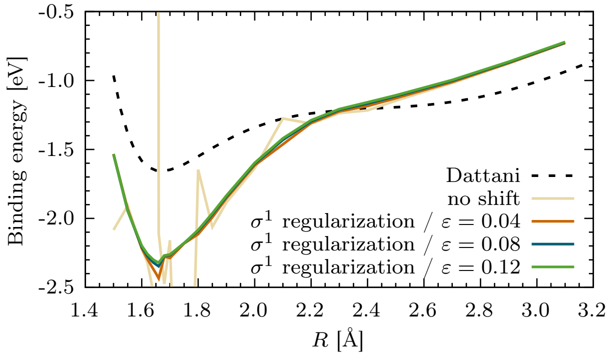

(a) Binding energy.

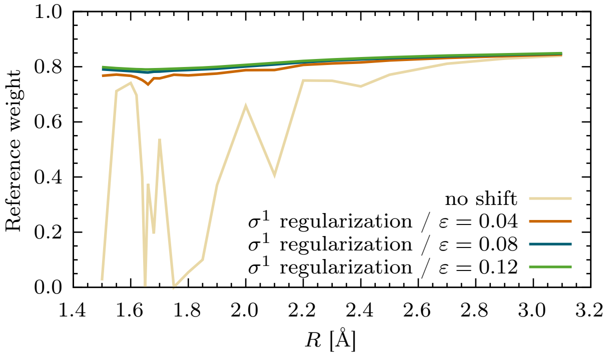

(b) Reference weight.

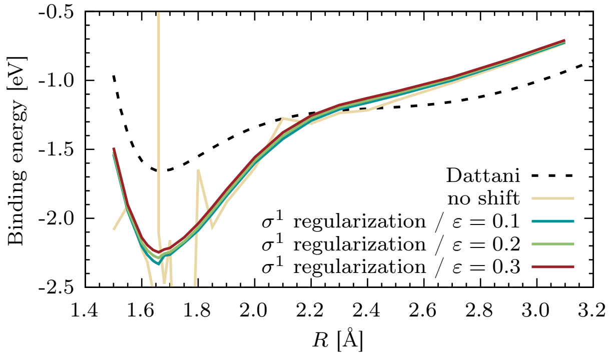

(c) Binding energy.

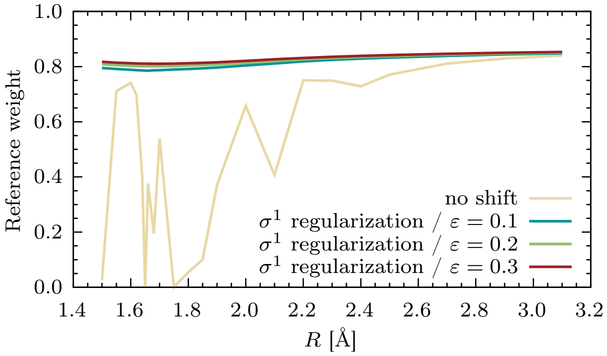

(d) Reference weight.

Figure S4: σ^1-CASPT2 potential energy curve of Cr_2 for different range of ε (a and c), and corresponding reference weight w_{ref} (b and d) as a function of the inter-nuclear distance R.

2.2 Sensitivity of σ^1-CASPT2 with respect to ε

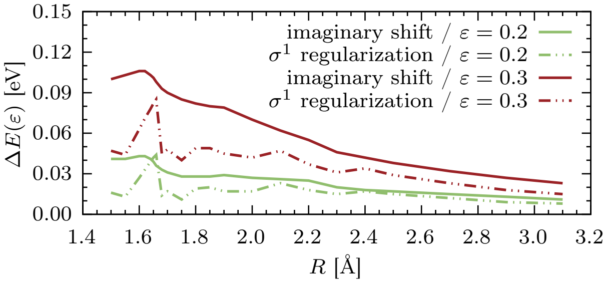

Figure S5: Energy difference $\Delta E(\varepsilon) = E(\varepsilon) - E_{\text{ref}}$ as a function of the inter-nuclear distance R, for two different values of ε. The reference energy E_{ref} used for each technique was obtained with the value $\varepsilon = 0.1\,E_{\text{h}}$. Note that the jump in energy for σ^1-CASPT2 is due to the discontinuity at $\Delta_i = 0$.

2.3 Potential energy curves for large values of ε

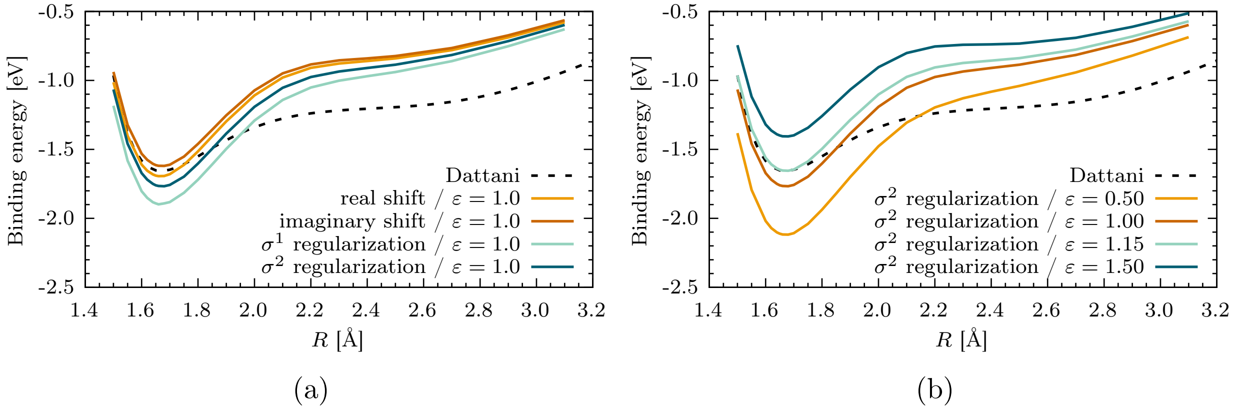

Figure S6: Potential energy curve of Cr_2 for the different techniques at the artificially large value of $\varepsilon = 1.0\,E_\mathrm{h}$ (a). σ^2-CASPT2 PEC for different large values of ε. For $\varepsilon = 1.15\,E_\mathrm{h}$, the calculated curve matches the experimental one around the equilibrium. However, at longer inter-nuclear distances the agreement is worse than with smaller values of ε.

2.4 Variational vs projected second-order energy

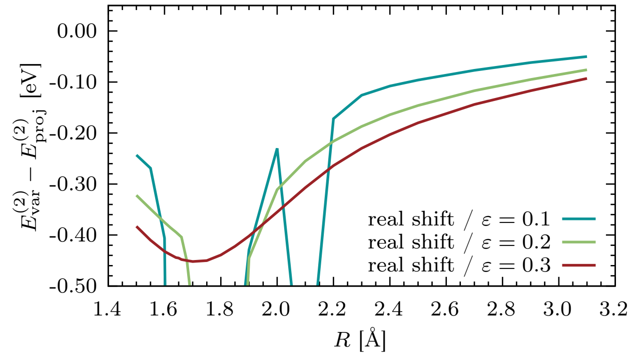

Figure S7: Difference between variational and projected second-order energy for the real level shift as a function of the inter-nuclear distance R.

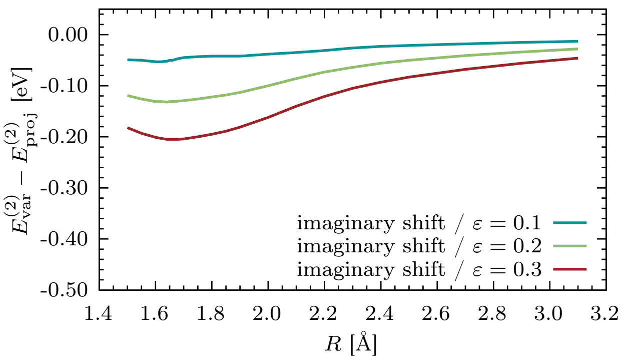

Figure S8: Difference between variational and projected second-order energy for the imaginary level shift as a function of the inter-nuclear distance R.

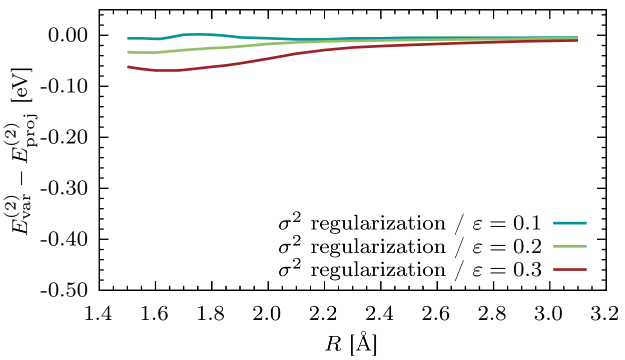

Figure S9: Difference between variational and projected second-order energy for σ^2-CASPT2 as a function of the inter-nuclear distance R.

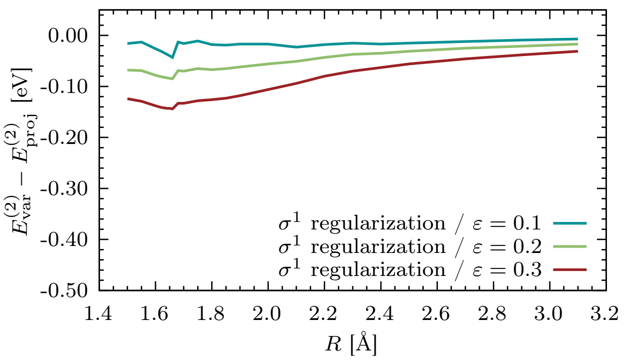

Figure S10: Difference between variational and projected second-order energy for σ^1-CASPT2 as a function of the inter-nuclear distance R.

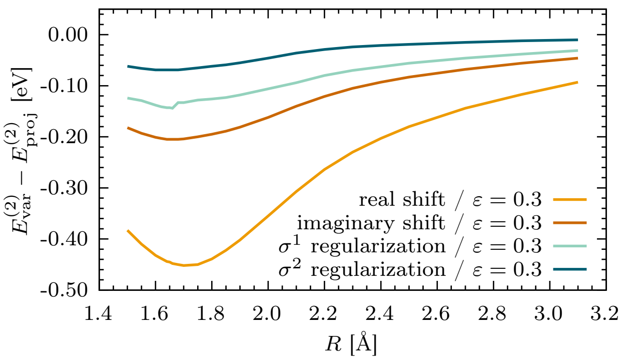

Figure S11: Difference between variational and projected second-order energy for $\varepsilon = 0.3\,E_\mathrm{h}$ as a function of the inter-nuclear distance R.

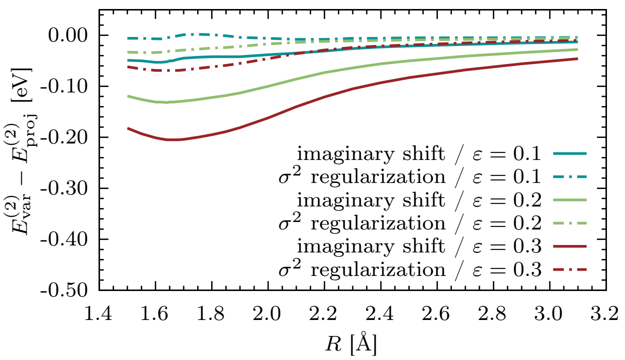

Figure S12: Direct comparison between the imaginary level shift and σ^2-CASPT2 of the difference between variational and projected second-order energy as a function of the inter-nuclear distance R.

3 Vertical excitation energies

3.1 Computational details

The details of the underlying SA-CASSCF calculations follow very closely those of Schreiber *et al.*[2] and can be found in their Supporting Information. In particular, the number of electronic states used in the state-averaging is exactly the same. The only differences are for the active spaces of four molecules. In imidazole, $5a'$ orbitals were used in the active space instead of (the erroneously reported) $6a'$. In pyridazine, $3a_2$ and $3b_1$ orbitals were used in the active space instead of $2a_2$ and $4b_1$. In pyridine, the active space used to compute the $^1B_2$ states is the same as the ground state one with 6 electrons in $4b_1$ and $2a_2$ orbitals, rather than with 8 electrons in $1a_1$, $8b_1$ and $4a_2$ orbitals. In tetrazine, 14 electrons were included in the active space instead of 12. All these differences are reported in the Supporting Information of the work by Zobel *et al.*[3]. Example inputs with these SA-CASSCF details (along with guess orbitals) are available for free in the OpenMolcas repository.[4]

Both the ISP and NOISP analyses were carried out with MS-CASPT2D and MS-CASPT2. An initial calculation without any shift was used to find states affected by intruder states. These were states that had a reference weight deviation larger than 10 % from the ground state weight (see Equation (23) in the manuscript). For MS-CASPT2D, there were 109 such states, which we report in Table S1, while for MS-CASPT2, there were 117 such states, which we report in Table S2.

Table S1: States excluded from the NOISP analysis for MS-CASPT2D.

Molecule	States excluded
acetamide	$2^3A''$
acetone	4^1A_1
adenine	$4–7^1A'$, $3–4^1A''$
benzene	$4–7^1A'$, $4–5^3A'$
benzoquinone	$2–3^1A_u$, $2–3^1B_{1g}$, $1–3^1B_{2g}$, 3^1B_{3g}, 4^1B_{1u}, $1–2^1B_{2u}$, $1–3^1B_{3u}$, 2^3A_u, 2^3B_{1g}
butadiene	3^1A_g
cyclopentadiene	$3–5^1A_1$
cyclopropene	—
cytosine	$4–6^1A'$
ethene	—
formaldehyde	3^1A_1
formamide	—
furan	3^1B_2, $3–4^1A_1$
hexatriene	1^1B_u
imidazole	$4–5^1A'$
naphthalene	$2–3^1B_{1g}$, $3,5^1B_{2u}$, $2–4^1B_{3u}$, $1–2^3A_g$, $2–3^3B_{1g}$, $2–3^1A_g$
norbornadiene	3^1A_1
octatetraene	$1,3^1B_u$, $3–4^1A_g$
propanamide	$2^3A''$
pyrazine	2^1B_{1g}, 2^1B_{2u}
pyridazine	2^1B_1, 4^1B_2, $3–4^1A_1$
pyridine	$2–3^1B_2$, 3^3A_1, 2^3B_2, 4^1A_1
pyrimidine	$2–4^1B_2$, 3^1A_1
pyrrole	3^1B_2, 3^1A_1
teatrazine	$2–3^1A_u$, $2–4^1B_{1g}$, 3^1B_{2g}, 3^1B_{3g}, 3^1B_{1u}, $3,5^1B_{2u}$, $2–3^1B_{3u}$, 2^3B_{1g}, 2^3B_{2g}, 2^3B_{3u}
thymine	$4–6^1A'$, $3–4^1A''$
triazine	$5–6^1A''$, $3–4,8^1A'$
uracil	$3–4^1A''$

Table S2: States excluded from the NOISP analysis for MS-CASPT2.

Molecule	States excluded
acetamide	2^3A$''$
acetone	4^1A$_1$
adenine	4–7^1A$'$, 3–4^1A$''$
benzene	4–7^1A$'$, 4–5^3A$'$
benzoquinone	3^1A$_u$, 2–3^1B$_{1g}$, 1–3^1B$_{2g}$, 3^1B$_{3g}$, 2,4^1B$_{1u}$, 1–2^1B$_{2u}$, 1–3^1B$_{3u}$, 2^3A$_u$, 2^3B$_{1g}$
butadiene	3^1A$_g$
cyclopentadiene	3–5^1A$_1$
cyclopropene	—
cytosine	4–6^1A$'$
ethene	—
formaldehyde	3^1A$_1$
formamide	2^3A$''$
furan	3^1B$_2$, 3–4^1A$_1$
hexatriene	1^1B$_u$
imidazole	2^3A$''$, 4–5^1A$'$
naphthalene	2–3^1B$_{1g}$, 3,5^1B$_{2u}$, 2–4^1B$_{3u}$, 1–2^3A$_g$, 2–3^3B$_{1g}$, 2–3^1A$_g$
norbornadiene	3^1A$_1$
octatetraene	1,3^1B$_u$, 3–4^1A$_g$
propanamide	2^3A$''$, 3^1A$'$
pyrazine	2^1B$_{1g}$, 2^1B$_{2u}$, 2^1A$_g$
pyridazine	2^1B$_1$, 3–4^1B$_2$, 3–4^1A$_1$
pyridine	2–3^1B$_2$, 3^3A$_1$, 2^3B$_2$, 4^1A$_1$
pyrimidine	2–4^1B$_2$, 4^1A$_1$
pyrrole	3^1B$_2$, 3^1A$_1$
teatrazine	2–3^1A$_u$, 1–4^1B$_{1g}$, 2–3^1B$_{2g}$, 3^1B$_{3g}$, 3^1B$_{1u}$, 3,5^1B$_{2u}$, 2–3^1B$_{3u}$, 2^3B$_{1g}$, 2^3B$_{2g}$, 2^3B$_{3u}$
thymine	4–6^1A$'$, 4^1A$''$
triazine	1,4–6^1A$''$, 3–4,8^1A$'$
uracil	3–4^1A$''$

3.2 ISP due to off-diagonal zeroth-order couplings

The second ^{1}B$_2$ state of pyrimidine has a reference weight deviation from the ground state of 15 % for CASPT2D and 25 % for CASPT2, hence it is considered an ISP in both cases. In

CASPT2D, the reference weight is a monotonic increasing function of the input parameter ε for both the imaginary shift and the two regularizers. This can be seen in Figures S13 to S15. In other words, it is certain that after a certain value of ε, the intruder state is removed.

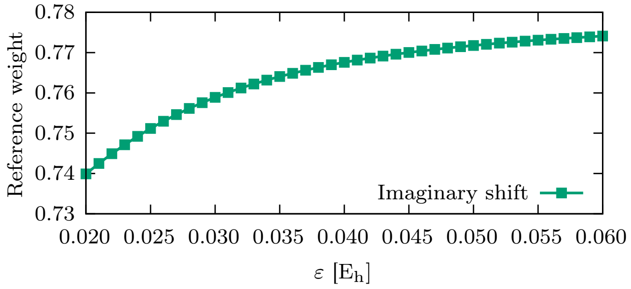

Figure S13: CASPT2D reference weight of the second $^1B_2$ of pyrimidine as a function of the imaginary level shift parameter.

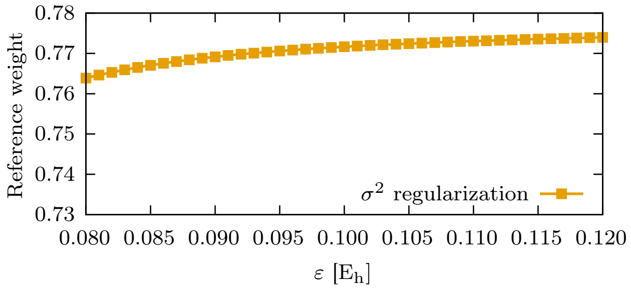

Figure S14: CASPT2D reference weight of the second $^1B_2$ of pyrimidine as a function of the σ^2 regularization parameter.

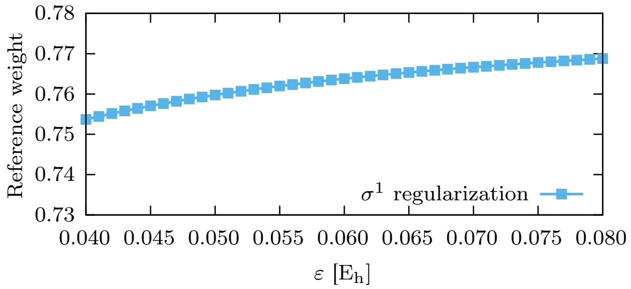

Figure S15: CASPT2D reference weight of the second $^1B_2$ of pyrimidine as a function of the σ^1 regularization parameter.

This is not the case when these techniques are used with a RSPT2 method that is not based on a diagonal zeroth-order Hamiltonian, such as CASPT2. Scanning the same range

of input parameters for the three methodologies reveals how a strong off-diagonal coupling leads to a singularity for a specific value of ε, as shown in Figures S16 to S18. Note that this value is specific to the intruder-state-removal technique used.

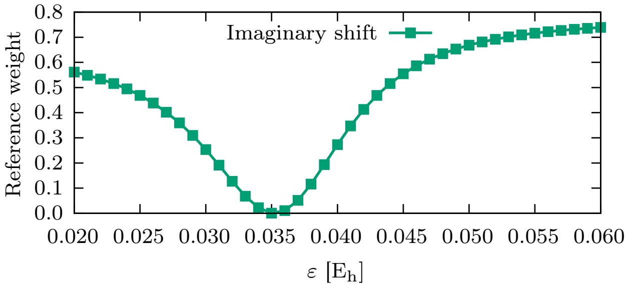

Figure S16: CASPT2 reference weight of the second $^1B_2$ of pyrimidine as a function of the imaginary level shift parameter.

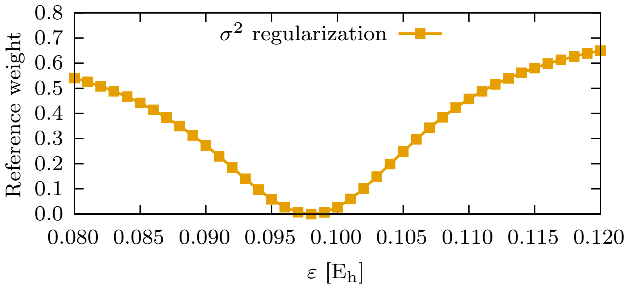

Figure S17: CASPT2 reference weight of the second $^1B_2$ of pyrimidine as a function of the σ^2 regularization parameter.

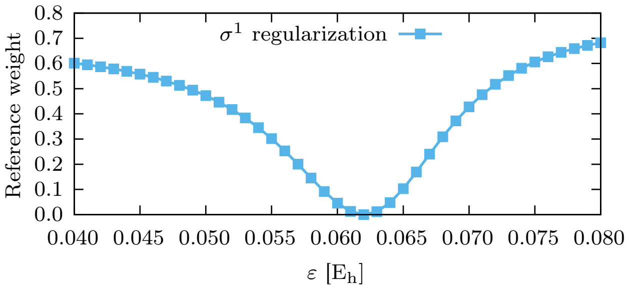

Figure S18: CASPT2 reference weight of the second $^1B_2$ of pyrimidine as a function of the σ^1 regularization parameter.

3.3 ISP analysis

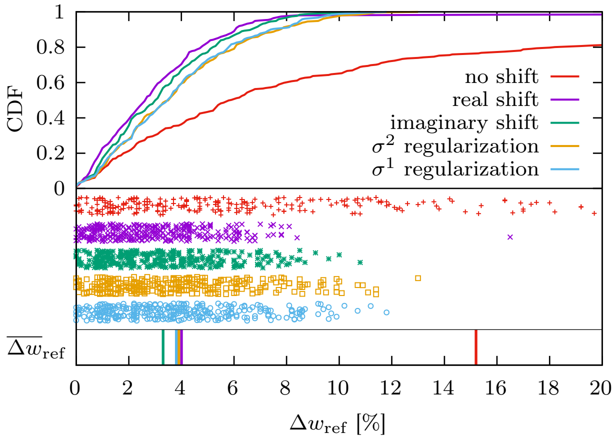

Figure S19: ISP analysis for CASPT2D with $\varepsilon = 0.2\,E_{\mathrm{h}}$. The cumulative distribution function is shown in the top half of the plots, while the values $\Delta w_{\mathrm{ref}} < 20\,\%$ are shown as points in the center of the plots. In the bottom stripe we show the position of the average reference weight deviation $\overline{\Delta w}_{\mathrm{ref}}$.

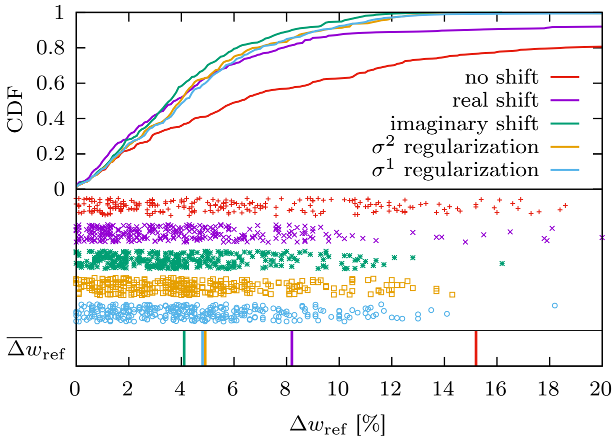

Figure S20: ISP analysis for CASPT2 with $\varepsilon = 0.1\,E_{\mathrm{h}}$. The cumulative distribution function is shown in the top half of the plots, while the values $\Delta w_{\mathrm{ref}} < 20\,\%$ are shown as points in the center of the plots. In the bottom stripe we show the position of the average reference weight deviation $\overline{\Delta w}_{\mathrm{ref}}$.

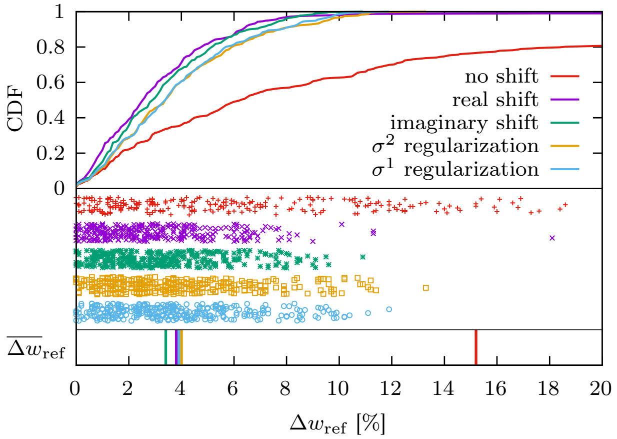

Figure S21: ISP analysis for CASPT2 with $\varepsilon = 0.2\,E_\text{h}$. The cumulative distribution function is shown in the top half of the plots, while the values $\Delta w_{\text{ref}} < 20\,\%$ are shown as points in the center of the plots. In the bottom stripe we show the position of the average reference weight deviation $\overline{\Delta w_{\text{ref}}}$.

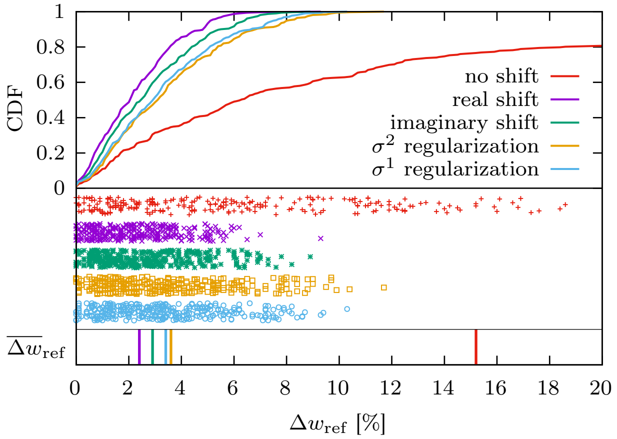

Figure S22: ISP analysis for CASPT2 with $\varepsilon = 0.3\,E_\text{h}$. The cumulative distribution function is shown in the top half of the plots, while the values $\Delta w_{\text{ref}} < 20\,\%$ are shown as points in the center of the plots. In the bottom stripe we show the position of the average reference weight deviation $\overline{\Delta w_{\text{ref}}}$.

References

(1) Roos, B. O.; Andersson, K. Multiconfigurational perturbation theory with level shift – the Cr_2 potential revisited. *Chem. Phys. Lett.* **1995**, *245*, 215–223.

(2) Schreiber, M.; Silva-Junior, M. R.; Sauer, S. P. A.; Thiel, W. Benchmarks for electronically excited states: CASPT2, CC2, CCSD, and CC3. *J. Chem. Phys.* **2008**, *128*, 134110.

(3) Zobel, J. P.; Nogueira, J. J.; González, L. The IPEA dilemma in CASPT2. *Chem. Sci.* **2017**, *8*, 1482–1499.

(4) OpenMolcas repository – CASPT2 benchmark. `https://gitlab.com/Molcas/OpenMolcas/-/tree/master/Tools/extra_tests/CASPT2_benchmark`, Accessed: 2022-04-11.
